# Respiratory Pathogen Co-Detection in Hospitalized Infants with Bronchiolitis and Implementation of Universal Nirsevimab Immunoprophylaxis: A Six-Year Observational Study

**DOI:** 10.3390/children13091189

**Published:** 2026-09-03

**Authors:** Gregorio Serra, Chiara Martorana, Giovanni Barbera, Mariavalentina Catania, Domenico Cipolla, Claudia Colomba, Piero Farruggia, Mario Giuffrè, Giulia La Malfa, Martina Greco, Giovanni Corsello

**Affiliations:** 1 Department of Health Promotion, Mother and Child Care, Internal Medicine and Medical Specialties “G. D’Alessandro”, University of Palermo, Via Alfonso Giordano 3, 90127 Palermo, Italy; giovanni.barbera01@unipa.it (G.B.); mario.giuffre@unipa.it (M.G.); giulia.lamalfa01@community.unipa.it (G.L.M.); giovanni.corsello@unipa.it (G.C.); 2 Children’s Hospital “Giovanni Di Cristina”, Azienda di Rilievo Nazionale e Alta Specializzazione Civico Di Cristina Benfratelli, Via dei Benedettini, 1, 90134 Palermo, Italy; chiara.martorana@arnascivico.it (C.M.); mariavalentina.catania@arnascivico.it (M.C.); domenico.cipolla@arnascivico.it (D.C.); claudia.colomba@unipa.it (C.C.); martina.greco@arnascivico.it (M.G.); 3 Unit of Pediatrics, Salvatore Cimino Hospital, Palermo Provincial Health Authority (ASP 6), Via Salvatore Cimino, 2, 90018 Termini Imerese, Italy; piero.farruggia@asppalermo.org

**Keywords:** respiratory syncytial virus, lower respiratory tract infections, viral–bacterial interactions, monoclonal antibodies, hospitalization

## Abstract

**Highlights:**

**What are the main findings?**

- Respiratory pathogen co-detection occurred in nearly half of infants hospitalized for bronchiolitis, and was associated with longer hospital stays and a higher rate of complications than single-virus infections.
- Respiratory syncytial virus (RSV) remained the predominant pathogen throughout the study period (2019–2025), while nirsevimab prophylaxis showed a favorable safety profile and was associated with lower RSV detection rates and fewer complications in the observational cohort.

**What are the implications of the main findings?**

- Respiratory pathogen co-detection may help recognize infants at increased risk of a more complex clinical course and optimize hospital management.
- These real-world findings support the feasibility and safety of implementing nirsevimab prophylaxis and suggest its potential to reduce the burden of RSV-related disease in early infancy, although confirmation from prospective studies is needed.

**Abstract:**

**Background:** Bronchiolitis is a leading cause of hospitalization in infants, with respiratory syncytial virus (RSV) as the main etiological agent. The clinical relevance of respiratory pathogen co-infections and the role of emerging preventive strategies, including nirsevimab immunoprophylaxis, remain under investigation. **Methods:** We conducted two complementary observational analyses within the pediatric healthcare network of Western Sicily. The first was a retrospective study including 580 children younger than 24 months hospitalized for bronchiolitis between 2019 and 2025 at the “Giovanni Di Cristina” Hospital in Palermo, Italy, to investigate respiratory pathogen detection patterns and their clinical correlates. Patients were classified according to microbiological findings as single-virus infections or viral–viral/viral–bacterial co-detections. The second was a separate observational cohort of 458 newborns from two birth centers in Western Sicily during the 2024–2025 RSV season, established to evaluate the implementation, safety, and respiratory outcomes associated with nirsevimab prophylaxis. **Results:** Viral–viral and/or viral–bacterial co-detections were identified in 45.5% of hospitalized infants and were associated with longer hospital stay (8.86 vs. 6.35 days, *p* < 0.001) and a higher frequency of complications (18.6% vs. 8.5%, *p* < 0.001), without differences in age, sex, or respiratory support requirements. RSV remained the predominant pathogen throughout the study period. In the complementary observational subgroup analysis, the observed safety findings were reassuring, with only mild and self-limiting events reported during follow-up; however, the sample size and observational design limit the ability to assess rare or delayed adverse events. Among infants undergoing clinically indicated respiratory testing, RSV was detected in 7/9 (77.8%) non-prophylaxed infants and 5/24 (20.8%) prophylaxed infants. Among hospitalized infants, complications were less frequently observed among those who had received nirsevimab (3.5% vs. 26.9%, *p* < 0.001), although substantial baseline differences between groups precluded causal interpretation of this finding. **Conclusions:** Viral–viral and viral–bacterial co-detections are frequent in hospitalized bronchiolitis and are associated with increased clinical complexity, mainly through prolonged hospitalization and higher occurrence of complications. The observational nirsevimab analysis showed a favorable safety profile and a lower proportion of RSV detection among clinically tested prophylaxed infants. However, because respiratory testing was performed according to clinical indication and differed substantially between groups, these findings cannot be interpreted as estimates of RSV incidence or as definitive evidence of nirsevimab effectiveness. The observed difference in complications should also be interpreted cautiously because of substantial baseline differences between prophylaxed and non-prophylaxed infants. Prospective studies with systematic respiratory testing are warranted.

## 1. Introduction

Bronchiolitis is an acute viral lower respiratory tract infection primarily affecting infants younger than two years of age and represents the leading cause of hospitalization in this population, particularly during the first year of life [1,2]. Clinical manifestations range from mild respiratory symptoms to severe respiratory distress, with a minority of infants progressing to acute respiratory failure requiring intensive respiratory support [1]. Respiratory syncytial virus (RSV) remains the predominant etiological agent, although other respiratory viruses, including rhinovirus, parainfluenza virus, human metapneumovirus, influenza viruses, and adenovirus, may be identified either as single pathogens or in combination with other microorganisms [2,3]. The increasing availability of multiplex molecular diagnostic techniques has substantially improved the detection of multiple respiratory pathogens, revealing that viral and viral–bacterial co-detections may occur among hospitalized infants with bronchiolitis [4,5]. However, the clinical significance of multiple pathogen detection remains incompletely understood. Previous studies have reported conflicting results regarding the association between coinfections and clinical outcomes, with some suggesting increased healthcare utilization, prolonged hospitalization, or more complex disease courses, whereas others have failed to demonstrate consistent differences compared with single-agent infections [5,6,7]. Furthermore, the interpretation of bacterial detection in respiratory samples remains challenging, as molecular identification of bacterial nucleic acids may reflect colonization or prolonged shedding rather than active infection, particularly for microorganisms commonly present in the upper respiratory tract [8]. Therefore, improving the understanding of the relationship between respiratory pathogen profiles and clinical evolution may contribute to better risk stratification and more appropriate management strategies in infants with bronchiolitis. Currently, treatment of bronchiolitis remains mainly supportive, as no widely available specific antiviral therapy exists [1]. Consequently, preventive strategies represent a cornerstone for reducing disease burden. RSV continues to have a major impact on pediatric healthcare because of its high transmissibility, seasonal circulation, and substantial contribution to infant hospitalizations worldwide [2,9]. The recent introduction of nirsevimab, a long-acting monoclonal antibody providing passive immunoprophylaxis against RSV, represents a major shift in prevention strategies, moving from targeted protection of selected high-risk infants toward a population-based approach targeting newborns and infants entering their first RSV season [10,11]. In Sicily, this transition was implemented for the first time during the 2024–2025 RSV season through a regional program established by the Sicilian Regional Health Department. Specifically, Regional Decree No. 725 of 11 June 2024, and subsequent Circular No. 30834 of 13 September 2024, introduced an active and free offer of nirsevimab to newborns and infants entering their first RSV season, with administration according to birth timing in relation to the RSV epidemic season [12]. Before this implementation, palivizumab was the only pharmacological tool available for RSV prevention in Italy and was administered selectively to infants at high risk of severe RSV disease [13,14].

The primary aim of this study was to evaluate the clinical and epidemiological characteristics of bronchiolitis requiring hospitalization in children younger than 24 months admitted to the “Giovanni Di Cristina” Pediatric Hospital in Palermo, Italy, over six epidemic seasons (2019–2025). This period included the pre-pandemic season, the disruption of respiratory virus circulation during the COVID-19 pandemic, the subsequent resurgence of respiratory pathogens, and the first season of universal RSV immunoprophylaxis with nirsevimab. Specifically, we compared clinical characteristics, management, and outcomes of bronchiolitis caused by single viral pathogens versus respiratory pathogen co-detections. We hypothesized that respiratory pathogen co-detections would be associated with greater clinical severity compared with single-virus infections. A complementary observational analysis was conducted during the 2024–2025 RSV season in two birth centers in Western Sicily, including the “Paolo Giaccone” University Hospital in Palermo and the “Salvatore Cimino” Hospital in Termini Imerese. This analysis aimed to describe the real-world implementation of universal nirsevimab prophylaxis, including adherence, safety profile, and clinical characteristics of prophylaxed and non-prophylaxed infants. We hypothesized that universal nirsevimab implementation would be feasible and well tolerated, and would be associated with a lower burden of respiratory illness and RSV detection among prophylaxed infants. Overall, this combined approach provides real-world insights into the evolving epidemiology of hospitalized bronchiolitis and the early implementation of a novel population-based RSV prevention strategy.

## 2. Materials and Methods

### 2.1. Study Design and Population

The present study comprised two distinct and complementary observational analyses conducted within the pediatric healthcare network of Western Sicily. The two analyses involved separate study populations, were conducted over different time periods, and addressed different objectives.

The first analysis was a retrospective observational study evaluating the epidemiological, microbiological, and clinical characteristics of hospitalized infants and young children with bronchiolitis over six consecutive epidemic seasons (2019–2020 to 2024–2025). Infants and children younger than 24 months of age presenting to the Pediatric Emergency Department of the “Giovanni Di Cristina” Hospital in Palermo, Italy, with clinical features consistent with acute bronchiolitis during the epidemic seasons, defined as October through March, were considered eligible for inclusion. Patients were included if they were subsequently admitted to the pediatric wards specializing in pneumology or infectious diseases at the same institution and received a clinical diagnosis of bronchiolitis. Bronchiolitis was diagnosed according to age-specific criteria consistent with international definitions [14], including acute onset of respiratory symptoms, respiratory distress, tachypnea, wheezing, and/or diffuse crackles on chest auscultation. For the purposes of the present study, microbiological characterization required the identification of at least one respiratory viral pathogen [1]. Exclusion criteria were age younger than 28 days, corresponding to patients managed within neonatal or neonatal intensive care units, and absence of microbiological identification. Patients in whom only a bacterial pathogen was detected, without evidence of viral detection, were also excluded because the study population was defined to reflect the predominantly viral microbiological profile of bronchiolitis. In contrast, patients with viral–bacterial co-detection were retained because the identification of a respiratory virus was consistent with the microbiological definition adopted for the study, while allowing the assessment of concomitant bacterial detection. This criterion was applied to maintain consistency between the clinical diagnosis and the microbiological classification of the study population. However, it precluded direct comparison between patients with isolated bacterial detection and those with viral–bacterial co-detection and should therefore be considered when interpreting the microbiological findings and the estimated burden of bacterial involvement.

The second analysis was conducted independently during the 2024–2025 RSV epidemic season and focused on the real-world implementation of RSV immunoprophylaxis with nirsevimab. The study population comprised newborns delivered at two hospitals providing maternity and neonatal care in Western Sicily: the “Paolo Giaccone” University Hospital in Palermo and the “Salvatore Cimino” Hospital in Termini Imerese. The eligibility period extended from November 2024 through 31 March 2025, corresponding to the period of the regional nirsevimab immunoprophylaxis campaign [12]. All eligible newborns delivered at the two centers during this period were offered nirsevimab according to the regional recommendations. At the “Paolo Giaccone” University Hospital, prophylaxis was initiated on 16 November 2024, and at the “Salvatore Cimino” Hospital on 27 November 2024; administration continued at both centers through 31 March 2025. Eligible infants included term and late-preterm newborns, as well as preterm infants and newborns with clinical conditions requiring admission to the neonatal intensive care unit or neonatal ward at the tertiary-level center. Nirsevimab was administered before discharge, generally between 48 and 72 h of life in clinically stable newborns. In infants requiring neonatal hospitalization, administration could be deferred until clinical stabilization and was performed before discharge. At the “Paolo Giaccone” University Hospital, families could also request administration after discharge through the neonatal follow-up clinic. Prophylaxis was offered on a voluntary basis following an information interview and written informed consent signed by both parents. The prophylaxed group included eligible infants who received nirsevimab, whereas the non-prophylaxed comparison group consisted exclusively of eligible infants whose parents declined prophylaxis. Nirsevimab was available throughout the study period; therefore, lack of drug availability or birth outside the active administration period did not account for non-prophylaxis. The two cohorts were analyzed separately and were not considered as a single study population. The objectives were to evaluate adherence to prophylaxis, safety outcomes, and respiratory outcomes during follow-up among infants who received nirsevimab compared with those who did not receive prophylaxis.

All data were collected from anonymized clinical records and entered into a dedicated electronic database.

### 2.2. Microbiological Assessment

Respiratory samples were obtained through nasopharyngeal swabs and/or hypopharyngeal aspirates collected under sterile conditions according to routine institutional procedures.

Total nucleic acids were extracted and analyzed using multiplex real-time polymerase chain reaction (PCR) assays targeting common respiratory pathogens. The primary diagnostic method was the Allplex™ Respiratory Full Panel assay (Seegene Inc., Seoul, Republic of Korea), which detects RSV A/B, influenza viruses, parainfluenza viruses, human metapneumovirus, adenovirus, rhinovirus/enterovirus, seasonal coronaviruses, SARS-CoV-2, and bacterial respiratory targets. Assays were performed according to the manufacturer’s instructions with appropriate internal quality controls. Conventional bacterial culture was performed when clinically indicated and according to routine laboratory workflow. Bacterial identification and antimicrobial susceptibility testing were performed following standard microbiological procedures and EUCAST criteria.

Because multiplex PCR detects bacterial nucleic acids and does not by itself distinguish viable organisms causing active infection from upper-airway colonization, bacterial molecular findings were not interpreted in isolation. Their clinical relevance was assessed in conjunction with the overall clinical and laboratory context, including inflammatory markers, blood cell parameters, clinical presentation, radiographic findings when indicated, and bacterial culture results when performed. A positive bacterial PCR result was therefore considered supportive of bacterial involvement rather than, by itself, definitive evidence of active bacterial infection. No validated quantitative PCR or cycle-threshold (Ct) cutoff was available or applied to distinguish colonization from active infection. Accordingly, the study did not use a specific molecular threshold to define bacterial infection. Viral–bacterial co-detection was classified according to the presence of both a respiratory virus and a bacterial molecular target, while the potential clinical significance of the bacterial detection was interpreted in the context of the accompanying clinical and laboratory findings. Detailed microbiological procedures are reported in Supplementary File S1.

### 2.3. Clinical Variables and Outcome Definitions

For each patient, the following variables were collected:

- demographic characteristics (exact age in months and sex);
- gestational age at birth and prematurity status (defined as gestational age < 37 weeks);
- presence of underlying comorbidities (e.g., congenital heart disease, chronic lung disease, neurological disorders, renal anomalies);
- identified viral and bacterial pathogens;
- length of hospital stay (LOS, in days);
- clinical characteristics at admission;
- oxygen requirement and respiratory support modalities (low-flow oxygen, high-flow nasal cannula [HFNC], non-invasive ventilation, invasive mechanical ventilation);
- admission to the Pediatric Intensive Care Unit (PICU);
- complications or bronchiolitis-associated conditions;
- pharmacological and supportive treatments;
- vaccination status;
- nirsevimab administration status (for the 2024–2025 season).

For the retrospective hospital-based analysis, the co-primary clinical endpoints were length of hospital stay and the occurrence of complications or bronchiolitis-associated conditions. The composite endpoint was defined as the occurrence of at least one predefined complication or bronchiolitis-associated condition during hospitalization. These conditions comprised:

- respiratory diseases (acute respiratory failure, pneumonia, bronchopneumonia, atelectasis, pleuritis, and pneumothorax);
- gastrointestinal disorders (gastroenteritis/enteritis);
- urinary tract infections;
- neurological events (seizures);
- other clinically relevant conditions recorded during hospitalization, including pericarditis, supraglottic laryngitis, gingivostomatitis, scabies, sepsis, cyanotic/apneic episodes, and heart failure with acute renal injury.

When more than one condition occurred in the same patient, individual events were recorded separately, whereas the patient was counted only once in the composite endpoint. To enhance clinical interpretability, individual components and their specific frequencies are reported separately in Supplementary File S2.

Secondary clinical endpoints included the requirement for supplemental oxygen, use of high-flow nasal cannula (HFNC), non-invasive ventilation, invasive mechanical ventilation, and PICU admission.

For the complementary 2024–2025 birth-center analysis, the primary outcomes were adherence to nirsevimab prophylaxis and safety outcomes during follow-up. Respiratory outcomes, including the occurrence of respiratory infections, RSV detection, medical evaluation or hospitalization, oxygen requirement, and clinical course among hospitalized infants, were considered secondary and exploratory outcomes.

### 2.4. Definition of Infection Groups

For the primary analysis, patients were classified according to microbiological findings into two main groups:

1. Single-virus infection group, i.e., patients with detection of a single viral pathogen.
2. Co-detection group, i.e., patients with simultaneous detection of two or more respiratory pathogens, including viral–viral and viral–bacterial combinations.

Patients with exclusive bacterial detection were excluded because bronchiolitis is predominantly a viral illness and, in the absence of viral detection, isolated bacterial detection was not considered consistent with the microbiological definition adopted for bronchiolitis in this study. This approach allowed the analysis to remain focused on clinically diagnosed bronchiolitis with documented viral involvement, while precluding direct comparison with patients with isolated bacterial detection.

To clarify whether clinical morbidity in the co-detection cohort was predominantly driven by bacterial co-pathogens versus viral–viral co-occurrence, the co-detection group was further stratified into two distinct subgroups:

2a viral–viral (V–V) co-detection, i.e., simultaneous detection of two or more viral pathogens without bacterial detection;

2b viral–bacterial (V–B) co-detection, i.e., simultaneous detection of at least one viral pathogen and one or more respiratory bacterial targets.

The main groups and subgroups were compared regarding demographic characteristics, clinical presentation, treatment requirements, hospitalization duration, and outcomes.

For the nirsevimab analysis, infants hospitalized or evaluated during the 2024–2025 RSV season were classified according to receipt of prophylaxis into prophylaxed and non-prophylaxed groups.

### 2.5. Statistical Analysis

A descriptive analysis was performed to summarize demographic, clinical, microbiological, and therapeutic characteristics of the study population. Categorical variables were expressed as frequencies and percentages, while continuous variables were reported as mean ± standard deviation or median with interquartile range (IQR), according to data distribution. Comparisons between patients with single-virus infections and those with co-detections, as well as between viral–viral and viral–bacterial subgroups, were performed using the chi-square test or Fisher’s exact test for categorical variables and the Student’s *t*-test or Mann–Whitney U test for continuous variables, as appropriate. The same analytical approach was applied to the subgroup analysis evaluating infants receiving nirsevimab prophylaxis during the 2024–2025 RSV season compared with non-prophylaxed infants. Stratified univariate analyses were implemented across clinical strata, specifically gestational age (preterm [<37 weeks] vs. term infants) and underlying comorbidities (presence vs. absence). To control for potential confounding variables, multivariate analyses were performed. A multivariate logistic regression model was used to evaluate the association between co-detection status and clinical complications, adjusting for age at admission, prematurity, underlying comorbidities, and epidemic season, reporting Adjusted Odds Ratios (aORs) with 95% Confidence Intervals (CIs). A multivariate linear regression model was fitted to assess the independent effect of co-detection on the length of hospital stay (days), adjusting for the same set of covariates and reporting regression coefficients (β) with 95% CIs. Statistical significance was defined as a two-sided *p*-value < 0.05.

## 3. Results

### 3.1. Study Population

Between the 2019–2020 and 2024–2025 epidemic seasons, a total of 580 infants younger than 24 months were hospitalized for bronchiolitis in the pediatric wards of the “Giovanni Di Cristina” Hospital in Palermo. The number of admissions varied across epidemic seasons, with 101 cases in 2019–2020, 16 in 2020–2021, 80 in 2021–2022, 133 in 2022–2023, 115 in 2023–2024, and 135 in 2024–2025.

Overall, 326/580 infants (56.2%) were male. The mean age at admission was 5.0 months (median 5.0 months; IQR, 1.5–7.0 months; range, 0.1–23.0 months), and the mean length of hospital stay was 7.5 ± 5.4 days (median 7 days; IQR, 4.0–8.0 days; range, 1–72 days). Complications or bronchiolitis-associated conditions were documented in 76 patients (13.1%), while comorbidities were identified in 74 patients (12.8%) and 46 infants (7.9%) were born preterm (<37 weeks gestational age) (for details see Supplementary File S2).

Regarding vaccination status, 200 infants (34.5%) were up to date according to the national immunization schedule, 201 (34.7%) were not vaccinated, 22 (3.8%) had delayed vaccinations, and vaccination status was unavailable in 157 cases (27.1%). During the 2024–2025 epidemic season, 57 of the 135 hospitalized infants (42.2%) had previously received nirsevimab prophylaxis.

Supportive and pharmacological treatments included oxygen supplementation, intravenous hydration, antibiotics, corticosteroids, bronchodilators, hypertonic saline, nebulized adrenaline, antifungal agents, and antiviral therapy. Detailed treatment regimens are reported in Supplementary File S2.

Based on microbiological findings, patients were classified into two groups: single-virus bronchiolitis (*n* = 316) and bronchiolitis associated with respiratory pathogen co-detection (*n* = 264). The distribution of cases according to epidemic season is summarized in Table 1.

### 3.2. Patients with Single-Virus Bronchiolitis

Among the 580 infants hospitalized for bronchiolitis, 316 (54.5%) had single-virus infections. Of these infants, 172 (54.4%) were male. The mean age at admission was 4.5 months (median 3.0 months; IQR, 1.0–6.0 months), and the mean duration of hospitalization was 6.35 days (median 5.0 days; IQR, 4.0–7.0 days).

RSV was the predominant pathogen throughout the study period, accounting for 208/316 cases (65.8%), followed by rhinovirus (45/316, 14.2%), SARS-CoV-2 (21/316, 6.6%), human metapneumovirus (20/316, 6.3%), influenza A (10/316, 3.2%), parainfluenza virus (7/316, 2.2%), influenza B (2/316, 0.6%), adenovirus (2/316, 0.6%), and coronavirus NL63 (1/316, 0.3%). The seasonal distribution of detected viral pathogens in the single-virus group is reported in Table 2.

Antibiotic therapy was administered to 142 infants (44.9%). Corticosteroids, bronchodilators, and supportive therapies were frequently used, while oxygen supplementation was required in 149 patients (47.2%). Low-flow oxygen was the most common respiratory support modality, whereas HFNC, continuous positive airway pressure (CPAP), and invasive mechanical ventilation were required in 4.5%, 0.3%, and 1.6% of cases, respectively (for details see Supplementary File S2).

Complications or associated conditions occurred in 27 infants (8.5%), mainly including respiratory failure, pneumonia, and bronchopneumonia. Comorbidities were identified in 40 patients (12.7%), with congenital heart disease representing the most frequent underlying condition. Seven infants experienced recurrent bronchiolitis, 17 (5.4%) had been born preterm, and 10 (3.2%) required admission to the PICU (Supplementary File S2).

### 3.3. Patients with Bronchiolitis and Respiratory Pathogen Co-Detection

Among the 580 infants hospitalized for bronchiolitis, 264 (45.5%) had respiratory pathogen co-detection. Of these infants, 154 (58.3%) were male. The mean age at admission was 5.0 months (median age 4.0 months; IQR, 2.0–8.0 months), and the mean length of hospital stay was 8.86 days (median 7.0 days; IQR, 5.0–10.0 days).

Among these patients, 67 cases (25.4%) involved viral–viral co-detections without bacterial identification, whereas 197 cases (74.6%) consisted of viral–bacterial co-detections. Because multiple pathogens could be detected in the same patient, the total number of viral and bacterial detections exceeded the number of enrolled infants. Therefore, percentages refer to the proportion of patients in whom each pathogen was identified rather than to the total number of microbiological detections.

RSV was the most frequently detected virus, identified in 184 patients (69.7%), followed by rhinovirus (100, 37.9%), human metapneumovirus (29, 11.0%), influenza A (19, 7.2%), adenovirus (18, 6.8%), parainfluenza virus (11, 4.2%), SARS-CoV-2 (6, 2.3%), and influenza B (6, 2.3%). Bacterial pathogens were detected in 273 instances, with *Haemophilus influenzae* (111 cases, 42.0%), *Streptococcus pneumoniae* (82, 31.1%), and *Moraxella catarrhalis* (31, 11.7%) being the most frequently identified organisms. The complete distribution of viral and bacterial detections in co-detection patients is summarized in Table 3. Granular visibility into specific dual- and multi-pathogen profiles (e.g., RSV + Rhinovirus, RSV + H. influenzae) is provided in Supplementary File S2 (Figure S1).

Antibiotic therapy was administered more frequently in the co-detection group than in the single-virus group (215/264, 81.4%). Oxygen supplementation was required in 120 patients (45.5%), mainly as low-flow oxygen therapy. HFNC, CPAP, and invasive mechanical ventilation were required in 3.0%, 0.4%, and 1.5% of cases, respectively (Supplementary File S2).

Complications or associated conditions occurred in 49 infants (18.6%), most frequently pneumonia, bronchopneumonia, and respiratory failure. Comorbidities were identified in 34 patients (12.9%), with congenital heart disease again representing the most common condition. Sixteen infants experienced recurrent bronchiolitis, 29 (11.0%) had been born preterm, and six (2.3%) required PICU admission (Supplementary File S2).

### 3.4. Comparison Between Single-Virus and Viral–Viral and/or Viral–Bacterial Co-Detection Bronchiolitis

Comparative analyses between the two groups are summarized in Table 4. Statistical analyses were performed using appropriate parametric or non-parametric tests according to variable distribution. Continuous variables are expressed as mean ± Standard Deviation (SD) and median (IQR). Categorical outcomes are reported as frequencies (%) with Odds Ratios (ORs) and 95% Confidence Intervals (CIs).

No significant differences were observed regarding sex distribution, age at admission, presence of comorbidities, respiratory support requirements, or invasive mechanical ventilation. Regarding the co-primary clinical endpoints, infants with viral–viral and/or viral–bacterial co-detection had a significantly longer hospitalization compared with those with single-virus bronchiolitis (mean length of stay 8.86 ± 7.42 days, median 7.0 [IQR, 5.0–10.0], vs. 6.35 ± 5.21 days, median 5.0 [IQR, 4.0–7.0], *p* < 0.001). Similarly, complications or bronchiolitis-associated conditions were more frequently observed among infants with co-detections (49/264, 18.6%) than among those with single-virus infections (27/316, 8.5%; OR = 2.44, 95% CI: 1.47–4.04; *p* < 0.001). Antibiotic therapy was more frequently administered in the co-detection group (81.4% vs. 44.9%; OR = 5.37, 95% CI: 3.65–7.91; *p* < 0.001), reflecting the higher prevalence of bacterial detection in this subgroup. PICU admission was uncommon in both groups (3.2% vs. 2.3%; OR = 0.71, 95% CI: 0.26–1.97; *p* = 0.780) and did not represent a clinically meaningful difference between cohorts.

#### 3.4.1. Subgroup Analysis of Co-Detections: Viral–Viral vs. Viral–Bacterial Co-Detection

To further investigate whether the greater clinical morbidity observed in the co-detection group differed according to the pathogen profile, a subgroup analysis comparing viral–viral (*n* = 67, 25.4%) and viral–bacterial (*n* = 197, 74.6%) co-detections was performed (Table 5a). Statistical significance was evaluated at a threshold of *p* < 0.05. Infants with viral–bacterial co-detection experienced a significantly longer median length of stay compared to those with viral–viral co-detection (mean length of stay 9.4 ± 7.8 days, median 8.0 days, IQR 6.0–10.0 vs. mean 7.2 ± 5.8 days, median 6.0 days, IQR 4.0–10.0; *p* = 0.008). Furthermore, complications were more frequent in the viral–bacterial subgroup than in the viral–viral subgroup (20.3% vs. 13.4%, OR = 1.64, 95% CI: 0.75–3.59; *p* = 0.210), and antibiotic administration was markedly higher in the viral–bacterial subgroup (90.9% vs. 53.7%, OR = 8.56, 95% CI: 4.32–16.96; *p* < 0.001). No significant differences were observed regarding male sex (58.9% vs. 56.7%; OR = 1.09, 95% CI: 0.62–1.94; *p* = 0.758), underlying comorbidities (13.2% vs. 11.9%; OR = 1.12, 95% CI: 0.48–2.61; *p* = 0.795), non-invasive oxygen support (46.2% vs. 43.3%; OR = 1.12, 95% CI: 0.64–1.97; *p* = 0.681), or PICU admission rates (2.5% vs. 1.5%; OR = 1.72, 95% CI: 0.20–15.01; *p* = 0.638).

#### 3.4.2. Stratified Analysis by Gestational Age and Comorbidities

To address potential confounding driven by baseline risk factors, stratified univariate analyses were conducted across clinical subgroups (statistical significance set at *p* < 0.05; Table 5b):

- By prematurity status. Among term infants (*n* = 534), length of stay remained significantly longer in the co-detection group compared with the single-virus group (median 7.0 days, IQR 5.0–10.0 vs. median 5.0 days, IQR 4.0–7.0; *p* < 0.001), and complications occurred significantly more frequently in co-detected infants (18.9% vs. 7.8%; OR = 2.73, 95% CI: 1.61–4.65; *p* < 0.001) In preterm infants (*n* = 46), length of stay was prolonged in both groups without reaching statistically significant differences (median 8.0 days, IQR 6.0–12.0 in co-detections vs. median 7.0 days, IQR 5.0–9.0 in single-virus; *p* = 0.665), nor did complication rates differ significantly between groups (16.7% vs. 30.0%; OR = 0.47, 95% CI: 0.09–2.32; *p* = 0.381), reflecting the baseline impact of prematurity on hospitalization length.
- By Underlying Comorbidities. Among infants without comorbidities (*n* = 506), co-detection remained significantly associated with longer hospitalization (*p* < 0.001) and higher complication rates (16.5% vs. 7.6%; OR = 2.40, 95% CI: 1.36–4.24; *p* = 0.002). In infants with underlying comorbidities (*n* = 74), median length of stay was extended in both groups (median 8.0 days vs. 7.0 days, *p* = 0.142), with a non-significant trend toward higher complication rates in co-detected infants (32.4% vs. 15.0%; OR = 2.71, 95% CI: 0.87–8.42; *p* = 0.081), suggesting that the association between co-detection and morbidity may persist beyond the contribution of baseline clinical risk factors.

**Table 5:** (**a**) Comparison of demographic characteristics, treatments, and clinical outcomes between viral–viral and viral–bacterial co-detection subgroups (n=264). Continuous variables are expressed as mean ± Standard Deviation (SD) and median (IQR). Categorical variables are reported as counts (%) and analyzed using the chi-square test or Fisher’s exact test, with Odds Ratios (ORs) and 95% Confidence Intervals (CIs). Significant *p*-values (<0.05) are in bold. (**b**) Stratified univariate analysis of hospital stay duration and complications by prematurity status and underlying comorbidities. Continuous variables compared using the Mann–Whitney U test; categorical variables compared using chi-square test or Fisher’s exact test with Odds Ratios (ORs) and 95% Confidence Intervals (CIs). Significant *p*-values (<0.05) are in bold.

(a)				
Variable	Viral–Viral Co-Detection (*n* = 67)	Viral–Bacterial Co-Detection (*n* = 197)	Odds Ratio (95% CI)	*p*-Value
Male sex, *n* (%)	38 (56.7%)	116 (58.9%)	1.09 (0.62–1.94)	0.758
Age at admission (months)				
—Mean ± SD	4.8 ± 3.9	5.1 ± 4.5	—	0.312
—Median [IQR]	3.5 [1.5–7.0]	4.0 [2.0–8.0]	—	
Length of hospital stay (days)				
—Mean ± SD	7.2 ± 5.8	9.4 ± 7.8	—	0.008
—Median [IQR]	6.0 [4.0–10.0]	8.0 [6.0–10.0]	—	
Complications/associated conditions, *n* (%)	9 (13.4%)	40 (20.3%)	1.64 (0.75–3.59)	0.210
Underlying comorbidities, *n* (%)	8 (11.9%)	26 (13.2%)	1.12 (0.48–2.61)	0.795
Antibiotic therapy, *n* (%)	36 (53.7%)	179 (90.9%)	8.56 (4.32–16.96)	**<0.001**
Non-invasive oxygen support, *n* (%)	29 (43.3%)	91 (46.2%)	1.12 (0.64–1.97)	0.681
PICU admission, *n* (%)	1 (1.5%)	5 (2.5%)	1.72 (0.20–15.01)	0.638
**(b)**				
**Clinical stratum**	**Single-virus bronchiolitis**	**Co-detection (v–v and v–b)**	**Odds Ratio (95% CI)**	***p*** **-value**
**By gestational age**				
Term Infants (≥37 weeks, *n* = 534)				
—Median length of hospital stay (days) [IQR]	5.0 [4.0–7.0]	7.0 [5.0–10.0]	—	**<0.001**
—Complications, *n* (%)	24/306 (7.8%)	43/228 (18.9%)	2.73 (1.61–4.65)	**<0.001**
Preterm Infants (<37 weeks, *n* = 46)				
—Median length of hospital stay (days) [IQR]	7.0 [5.0–9.0]	8.0 [6.0–12.0]	—	0.665
—Complications, *n* (%)	3/10 (30.0%)	6/36 (16.7%)	0.47 (0.09–2.32)	0.381
**By underlying comorbidities**				
No underlying comorbidities (*n* = 506)				
—Median length of hospital stay (days) [IQR]	5.0 [4.0–7.0]	7.0 [5.0–10.0]	—	**<0.001**
—Complications, *n* (%)	21/276 (7.6%)	38/230 (16.5%)	2.40 (1.36–4.24)	0.002
Presence of underlying comorbidities (*n* = 74)				
—Median length of hospital stay (days) [IQR]	7.0 [4.0–9.0]	8.0 [5.0–12.0]	—	0.142
—Complications, *n* (%)	6/40 (15.0%)	11/34 (32.4%)	2.71 (0.87–8.42)	0.081

CI: confidence interval; IQR: interquartile range; OR: odds ratio; PICU: pediatric intensive care unit; SD: standard deviation. CI: confidence interval; IQR: interquartile range; OR: odds ratio; v–v: viral–viral; v–b: viral–bacterial.

#### 3.4.3. Multivariate Regression Analysis

To further isolate the independent effect of pathogen co-detection on clinical outcomes, multivariate regression models were conducted, adjusting for age at admission, prematurity status, underlying comorbidities, and epidemic season:

- Complications (multivariate logistic regression): respiratory pathogen co-detection remained an independent predictor of bronchiolitis-associated complications (adjusted Odds Ratio, aOR = 2.15, 95% CI: 1.25–3.69; *p* = 0.006). Older age at admission was also independently associated with higher complication rates (aOR = 1.09 per month of age, 95% CI: 1.04–1.15; *p* = 0.001), whereas prematurity and underlying comorbidities did not retain statistically independent associations in the adjusted model.
- Length of hospital stay (multivariate linear regression): co-detection was independently associated with a significantly prolonged hospital stay, contributing an additional +2.37 days of hospitalization (adjusted β = 2.37, 95% CI: 1.56–3.18; *p* < 0.001). The presence of underlying comorbidities was also an independent driver of extended hospitalization (adjusted β = 2.65 days, 95% CI: 1.48–3.81; *p* < 0.001).

These findings support an independent association between respiratory pathogen co-detection and prolonged hospital stay and bronchiolitis-associated complications, after adjustment for the baseline factors included in the models.

### 3.5. Focus on Nirsevimab Prophylaxis (2024–2025 Season)

Given the introduction of nirsevimab during the 2024–2025 RSV season [14], a dedicated observational analysis was performed among the 135 infants hospitalized for bronchiolitis during the 2024–2025 season, of whom 57 (42.2%) had received nirsevimab and 78 (57.8%) had not. Importantly, this proportion should not be interpreted as the proportion of infants requiring hospitalization despite nirsevimab at the population level. Rather, the 57 prophylaxed infants represent a selected group of hospitalized infants who developed bronchiolitis despite having received prophylaxis, including breakthrough RSV infections and cases in which a non-RSV viral pathogen was identified. Thus, this cohort represents a hospital-based breakthrough/clinically affected population rather than a population-based estimate of nirsevimab effectiveness. This is fundamentally different from the cohort of newborns evaluated at birth centers (see the following section), which represents a broader population receiving nirsevimab for primary prevention and is therefore more appropriate for assessing population-level preventive effectiveness.

Complications or bronchiolitis-associated conditions were observed in 2/57 (3.5%) prophylaxed infants and 21/78 (26.9%) non-prophylaxed infants (OR = 0.10, 95% CI: 0.02–0.44; *p* < 0.001), while mean hospital stay was 5.6 ± 2.1 days (median 5.0 [IQR, 4.0–6.0]) versus 7.0 ± 4.3 days (median 6.0 [IQR, 4.0–8.0]), respectively (*p* = 0.025). Mean age at admission was 4.4 ± 3.2 months (median 3.0 [IQR, 1.0–5.0]) in the nirsevimab group versus 6.2 ± 4.8 months (median 5.0 [IQR, 2.0–9.0]) in the non-prophylaxed group (*p* = 0.005). Given the baseline imbalance between groups, these differences should be interpreted as descriptive associations and should not be considered evidence of a causal protective effect of nirsevimab.

No statistically significant differences were observed between groups regarding antibiotic therapy, need for non-invasive respiratory support, or distribution of single-virus versus co-detection bronchiolitis. No infant required invasive mechanical ventilation, and only one patient, belonging to the non-prophylaxed group, required pediatric intensive care admission. A summary of comparative analyses is provided in Table 6.

### 3.6. Real-World Implementation of Nirsevimab Prophylaxis (2024–2025 Season)

A complementary multicenter observational analysis was performed to evaluate the real-world implementation of universal nirsevimab prophylaxis during the 2024–2025 RSV season. The study included 458 neonates born at two participating centers in Western Sicily: the Neonatology Unit of the “Paolo Giaccone” University Hospital in Palermo (*n* = 291, 63.5%) and the “Salvatore Cimino” Hospital in Termini Imerese (*n* = 167, 36.5%).

Of the 458 newborns included in the nirsevimab cohort, follow-up information was available for 399 (87.1%). The remaining 59 infants (12.9%) could not be reached despite repeated attempts to obtain follow-up information by online questionnaire (Supplementary File S3) and subsequent telephone contact, and were therefore excluded from the follow-up analysis concerning post-discharge outcomes. Nirsevimab administration was documented in 415 infants, whereas 43 infants did not receive prophylaxis. For analyses requiring both follow-up information and a documented prophylaxis status, the available denominators varied according to the specific outcome.

The overall mean gestational age was 38.7 ± 2.1 weeks (median 39 weeks). Infants born at the University Hospital had a lower mean gestational age compared with those born at the peripheral center (38.3 vs. 39.3 weeks; *p* < 0.001), with a higher proportion of preterm births (15.2% vs. 2.4%; *p* < 0.001). Mean birth weight was also lower among infants born at the University Hospital (3070 g vs. 3247 g), whereas the distribution according to birth weight for gestational age (appropriate, small, or large for gestational age) was comparable between centers.

**Figure 1:**
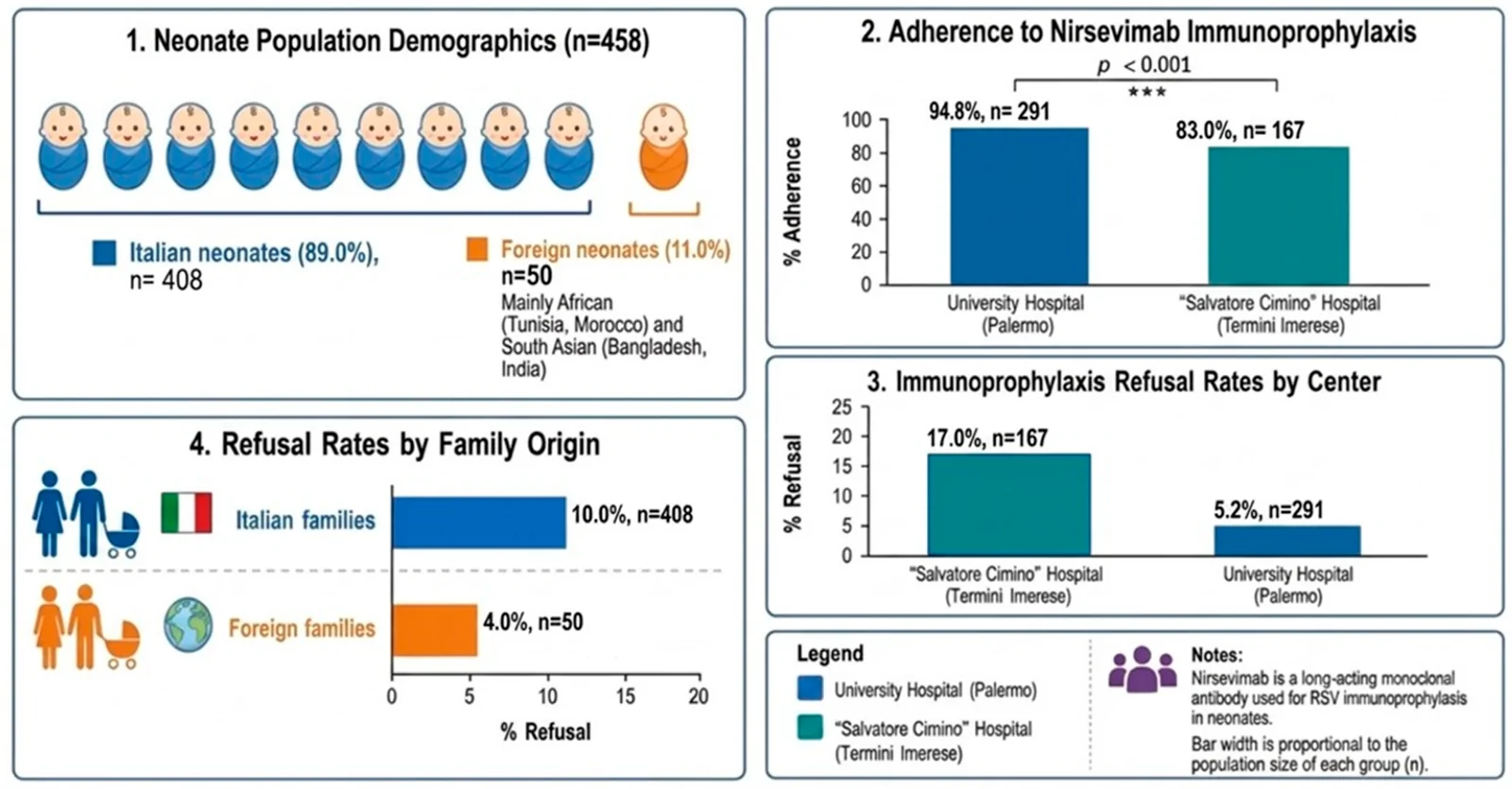
Adherence and refusal rates for nirsevimab prophylaxis according to study center and nationality. Adherence was higher at the University Hospital (94.8%, n=276/291) than at the peripheral center (83.0%, n=139/167), with corresponding refusal rates of 5.2% (n=15) and 17.0% (n=28) (*** p<0.001). By nationality, Italian families (89.1%, n=408) had a 10.0% refusal rate (n=41), compared with 4.0% (n=2) among foreign families (10.9%, n=50). Bar width is proportional to group size.

Overall, 89.0% (n=408/458) of infants were born to Italian families and 11.0% (n=50/458) to foreign families. Nirsevimab uptake was high, although differences were observed between centers. Prophylaxis was administered to 94.8% (n=276/291) of infants at the University Hospital and to 83.0% (n=139/167) at the peripheral center (p<0.001). Consequently, refusal rates were higher at the peripheral center (17.0%, n=28/167 vs. 5.2%, n=15/291). Refusal was also more frequent among Italian families compared with foreign families (10.0%, n=41/408 vs. 4.0%, n=2/50, respectively). Adherence patterns and refusal rates according to healthcare setting and nationality are illustrated in Figure 1.

The timing of prophylaxis administration differed between centers (*p* = 0.020). At the peripheral center, most infants received nirsevimab within the first days of life, whereas administration at the University Hospital was more frequently postponed until clinical stabilization or discharge, particularly among preterm or medically complex neonates.

### 3.7. Safety and Tolerability of Nirsevimab

Safety outcomes were actively solicited by the investigators during follow-up. Families were specifically asked about adverse events after nirsevimab administration, including fever and local injection-site reactions, using the predefined follow-up questionnaire (Supplementary File S3). When the questionnaire was not completed online, the same information was obtained by telephone interview. Follow-up was conducted between July and August 2025, with a variable interval between nirsevimab administration and follow-up according to the date of prophylaxis. Among the 415 infants who received nirsevimab prophylaxis, adverse events were reported in 13 cases (3.1%). All reported events were mild, transient, and self-limiting. Injection-site erythema and swelling were observed in 4 infants each (1.0%), while low-grade fever occurred in 5 infants (1.2%) (Table 7).

No infant required additional medical evaluation, specific treatment, or hospitalization because of adverse events. Among infants reporting adverse events, 1 had been born preterm and small for gestational age, 2 were term infants classified as small for gestational age, while the remaining cases had no relevant perinatal risk factors.

### 3.8. Respiratory Outcomes Following Nirsevimab Prophylaxis

During follow-up, respiratory tract infections were reported in 147 of 399 infants (36.8%). The proportion of respiratory infections was higher among infants born at the University Hospital than among those born at the peripheral center (40.2% vs. 31.3%), although the difference was not statistically significant (*p* = 0.090). Respiratory infections were associated with feeding modality. Exclusively breastfed infants showed the lowest frequency of respiratory infections (26.2%) compared with infants receiving mixed feeding (39.5%) or exclusive formula feeding (43.3%; *p* = 0.009). No exclusively breastfed infant had laboratory-confirmed RSV detection, whereas RSV detection was observed among mixed-fed and formula-fed infants (4.0% and 3.5%, respectively; *p* = 0.007). The presence of older siblings was associated with a higher frequency of respiratory infections (69.9% vs. 49.8% among only children; *p* < 0.001), although no significant association was observed specifically with RSV detection.

Among infants with available data for this analysis, respiratory infections occurred in 18/39 (46.2%) non-prophylaxed infants compared with 129/359 (35.9%) infants who received nirsevimab; this difference was not statistically significant (*p* = 0.300). Among infants requiring medical evaluation for respiratory symptoms, most cases were managed by primary care pediatricians (72.8%), whereas 9.6% required emergency department assessment and 17.7% hospitalization. Hospital admission for lower respiratory tract infections occurred more frequently among non-prophylaxed infants, although this difference did not reach statistical significance (*p* = 0.200). Among the 26 hospitalized infants, length of stay was shorter among those who had received nirsevimab than among non-prophylaxed infants (mean 5.3 vs. 29.8 days; median 4 vs. 10 days; *p* = 0.010). No significant differences were observed in oxygen requirement between groups (35.7% vs. 28.6%; *p* = 0.990).

### 3.9. RSV Detection According to Nirsevimab Prophylaxis

**Figure 2:**
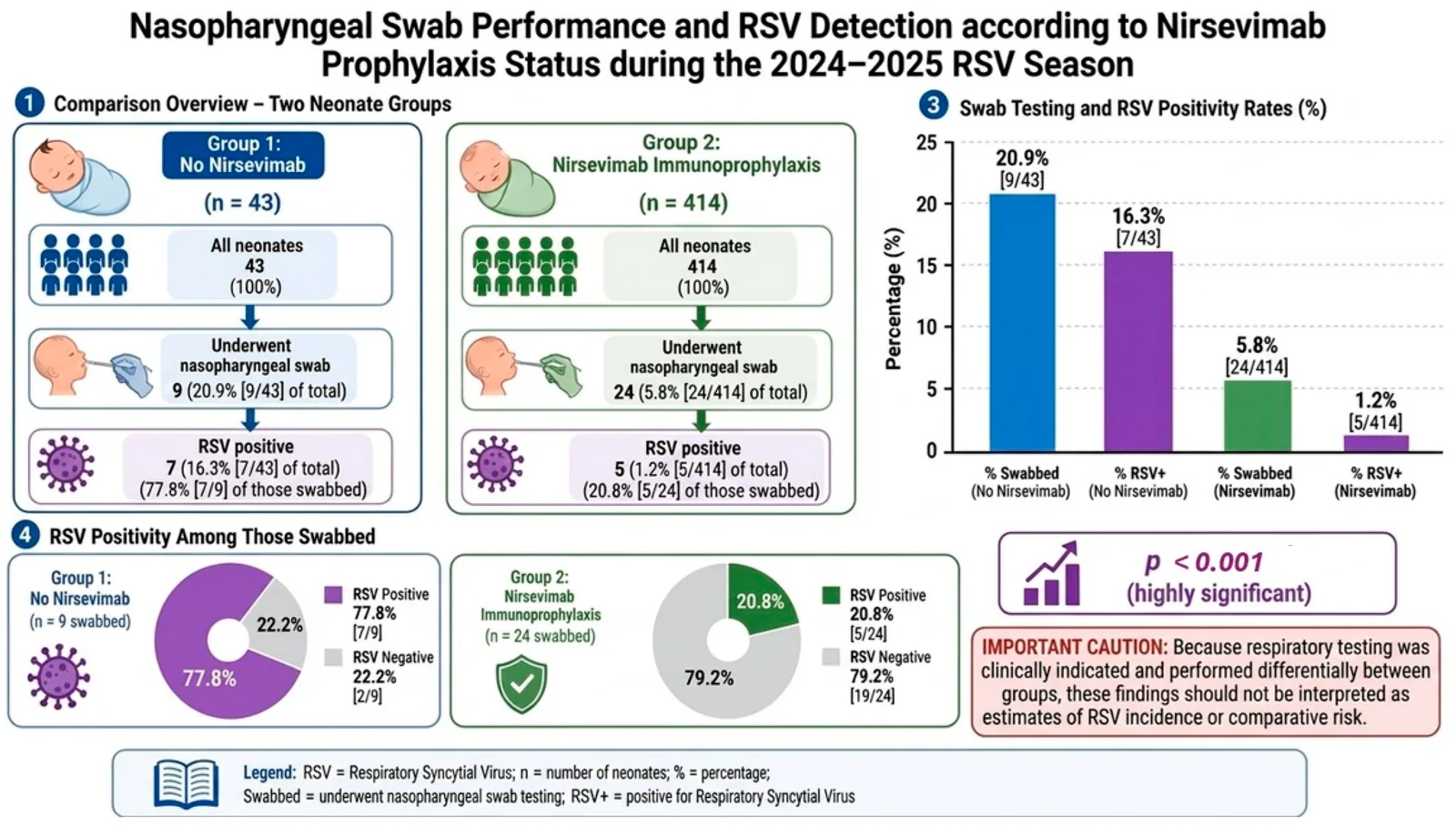
Nasopharyngeal swab performance and RSV detection according to nirsevimab prophylaxis status during the 2024–2025 RSV season. Respiratory sampling was performed more frequently among non-prophylaxed infants than among those who received nirsevimab (20.9% vs. 5.8%). Among clinically tested infants, RSV was detected in 7/9 (77.8%) non-prophylaxed infants and 5/24 (20.8%) prophylaxed infants (*p* < 0.001). When calculated relative to the total number of infants in each prophylaxis group, RSV detection was 16.3% (7/43) and 1.2% (5/414), respectively. Because respiratory testing was clinically indicated and performed differentially between groups, these findings should not be interpreted as estimates of RSV incidence or comparative risk.

Nasopharyngeal swabs for respiratory viruses were performed more frequently among non-prophylaxed infants than among those who received nirsevimab (9/43 [20.9%] vs. 24/414 [5.8%]). Among infants who underwent respiratory testing, RSV was detected in 7/9 (77.8%) non-prophylaxed infants and in 5/24 (20.8%) prophylaxed infants (*p* < 0.001). When expressed relative to the total number of infants in each prophylaxis group, RSV was detected in 7/43 (16.3%) non-prophylaxed infants and 5/414 (1.2%) prophylaxed infants. However, respiratory testing was performed according to clinical indication rather than systematically, and was substantially more frequent among non-prophylaxed infants. Therefore, these findings should be interpreted as descriptive measures of RSV detection, and should not be considered estimates of RSV incidence or comparative RSV risk in the overall prophylaxed and non-prophylaxed populations. Differences in respiratory virus testing and RSV detection according to prophylaxis status are shown in Figure 2.

When the two participating centers were analyzed separately, RSV-positive swabs were more frequently detected at the Termini Imerese Hospital than at the University Hospital (4.8% vs. 1.4%, *p* = 0.040). In both settings, RSV positivity remained consistently lower among infants who had received nirsevimab prophylaxis. Gestational age did not differ significantly between prophylaxed and non-prophylaxed infants (38.6 vs. 39.2 weeks; *p* = 0.200), and none of the infants with confirmed RSV detection had been born preterm.

## 4. Discussion

The present study provides a comprehensive overview of the epidemiology and clinical characteristics of bronchiolitis over six consecutive epidemic seasons, encompassing the pre-pandemic period, the disruption of respiratory virus circulation during the COVID-19 pandemic, the subsequent resurgence of respiratory pathogens, and the first season of universal RSV immunoprophylaxis with nirsevimab. Three main findings emerge from our analysis. First, viral–viral and viral–bacterial co-detections were common, affecting nearly half of hospitalized infants. Second, co-detections were associated with greater clinical complexity, as reflected by longer hospital stays and a higher frequency of complications, although not by an increased need for respiratory support. Third, the first real-world experience with universal nirsevimab administration in Western Sicily demonstrated high parental acceptance, an excellent safety profile, and clinical findings consistent with the growing evidence supporting RSV immunoprophylaxis.

In the 2024–2025 season, 57 of 135 hospitalized infants (42.2%) had received nirsevimab. This proportion should not be interpreted as evidence of low effectiveness of RSV immunoprophylaxis, as hospitalization for bronchiolitis after nirsevimab administration does not necessarily represent prevention failure against RSV. Nirsevimab specifically targets RSV and does not provide protection against other respiratory viruses; therefore, a proportion of these hospitalized infants had bronchiolitis caused by pathogens such as rhinovirus, adenovirus, or human metapneumovirus, which are unaffected by RSV-specific immunoprophylaxis. In addition, the occurrence of RSV-associated hospitalization despite prophylaxis may reflect incomplete or time-dependent protection, individual differences in exposure and susceptibility, or high RSV transmission pressure during the epidemic season. Importantly, the proportion of nirsevimab-exposed infants among hospitalized patients cannot be used to estimate real-world effectiveness, as an appropriate effectiveness assessment would require comparison with the incidence of RSV hospitalization in a comparable unprotected population and adjustment for timing of administration, age, underlying risk factors, and circulating RSV burden. Thus, these findings should be interpreted primarily as a description of breakthrough hospitalizations occurring during the first season of widespread nirsevimab implementation rather than as a measure of prophylactic failure.

Viral–viral and viral–bacterial co-detections were identified in 45.5% of hospitalized children, a prevalence consistent with previous studies reporting rates ranging from 30% to 50% among infants investigated with multiplex molecular assays [4]. The widespread adoption of multiplex PCR has substantially improved the detection of respiratory pathogens, revealing a greater frequency of mixed viral and viral–bacterial infections than previously recognized. However, the detection of bacterial nucleic acids in upper respiratory tract specimens should be interpreted cautiously, as molecular positivity may reflect colonization rather than active infection, particularly for microorganisms that commonly inhabit the nasopharynx [15]. Consequently, microbiological findings should always be interpreted within the broader clinical context.

Interestingly, demographic characteristics were comparable between children with single-virus infections and those with co-detections. Neither age, sex, nor the prevalence of underlying comorbidities differed significantly between the two groups, suggesting that the occurrence of multiple pathogen detection is more likely related to the epidemiological dynamics of respiratory pathogen circulation than to baseline patient characteristics. Although prematurity, congenital heart disease, and chronic respiratory disorders are well-established risk factors for severe bronchiolitis [16], our findings indicate that the increased clinical burden associated with microbiological co-detections may also occur in infants without major pre-existing medical conditions.

The principal finding of our study is the association between viral–viral and viral–bacterial co-detections and a more complex clinical course. Infants with multiple pathogen detections experienced substantially longer hospitalizations and more frequently developed complications than those with single-virus bronchiolitis. Importantly, these associations remained evident after adjustment for relevant baseline factors. In the multivariate logistic regression model, respiratory pathogen co-detection was independently associated with an increased likelihood of complications (adjusted Odds Ratio [aOR] = 2.15, 95% CI: 1.25–3.69; *p* = 0.006), after adjustment for age at admission, prematurity, underlying comorbidities, and epidemic season. Similarly, in the multivariate linear regression model, co-detection was independently associated with an additional 2.37 days of hospitalization (adjusted β = 2.37, 95% CI: 1.56–3.18; *p* < 0.001). Underlying comorbidities were also independently associated with prolonged hospitalization (adjusted β = 2.65 days, 95% CI: 1.48–3.81; *p* < 0.001). These findings strengthen the interpretation that pathogen co-detection represents an independent marker of a more prolonged and clinically complicated course, beyond the contribution of the baseline factors included in the models. To further refine the interpretation of this finding and establish whether clinical morbidity was driven by specific microbiological combinations or influenced by baseline patient vulnerability, dedicated subgroup and stratified analyses were performed. When dissecting the co-detection cohort (Table 5a), infants with viral–bacterial co-detections exhibited a significantly longer median length of stay compared to those with viral–viral co-detections (8.0 vs. 6.0 days, *p* = 0.008), alongside higher antibiotic usage (90.9% vs. 53.7%, *p* < 0.001). These findings suggest that bacterial detection identifies a subgroup of infants with greater healthcare utilization and a more prolonged hospital course. However, this result should not be interpreted as evidence that bacterial co-pathogens directly caused the increased morbidity, particularly because molecular detection cannot reliably distinguish active bacterial infection from upper-airway colonization. Furthermore, complications were numerically more frequent in the viral–bacterial subgroup than in the viral–viral subgroup (20.3% vs. 13.4%), but this difference did not reach statistical significance (*p* = 0.210). Furthermore, to address potential confounding by baseline clinical risk factors, univariate stratified analyses were executed across key clinical strata (Table 5b). Importantly, the significant association between pathogen co-detection and both prolonged hospitalization (*p* < 0.001) and elevated complication rates (*p* < 0.001) persisted among term infants (≥37 weeks) and infants without underlying comorbidities. In contrast, among preterm infants (<37 weeks) and those with pre-existing conditions, hospitalization duration was overall prolonged regardless of microbiological profile (median 7.0–8.0 days across sub-groups), and the differences between co-detection and single-virus groups did not reach statistical significance. These findings suggest that prematurity and underlying medical conditions may exert a substantial influence on clinical course, and may reduce the ability to detect an additional effect associated with pathogen co-detection in these higher-risk subgroups. The absence of statistical significance should nevertheless be interpreted cautiously, particularly given the relatively small number of preterm infants and infants with comorbidities. Taken together, the consistency between the overall comparisons, stratified analyses, and multivariate models supports an association between respiratory pathogen co-detection and increased clinical morbidity. Although co-detection was independently associated with complications and prolonged hospitalization after adjustment for age, prematurity, comorbidities, and epidemic season, the observational design of the study precludes establishing a causal relationship, and residual confounding cannot be completely excluded. Although co-detections were not associated with greater respiratory support requirements in our cohort, the absence of significant differences in oxygen requirements, invasive mechanical ventilation, or PICU admission indicates that co-detection was not associated with all markers of acute respiratory severity. Rather, its clinical relevance in our cohort appears to be more closely related to prolonged hospitalization and the occurrence of bronchiolitis-associated complications than to escalation of respiratory support.

Several biological mechanisms could potentially contribute to the observed associations, although these mechanisms cannot be established from our data. In viral–bacterial interactions, primary viral infection may cause epithelial injury, disrupt mucosal barrier integrity, impair mucociliary clearance, and alter local innate immune responses, thereby potentially facilitating bacterial adherence, persistence, and proliferation and increasing the risk of secondary bacterial complications [17,18]. In contrast, during viral–viral co-infections, interactions may depend on the timing and biological characteristics of the viruses involved, including competition for susceptible epithelial cells and induction of antiviral states that may interfere with or facilitate replication of a second virus [19]. Simultaneous or sequential viral infections may also be accompanied by enhanced inflammatory responses and cumulative epithelial injury, potentially contributing to a more prolonged clinical course [20]. These mechanisms provide biologically plausible explanations for the observed associations but should not be interpreted as evidence of causality in the present study [21,22]. Furthermore, because molecular assays cannot reliably distinguish active bacterial infection from upper-airway colonization, particularly for organisms such as *Haemophilus influenzae* and *Streptococcus pneumoniae*, the individual contribution of each detected microorganism to disease severity cannot be established with certainty. Accordingly, pathogen co-detection should be regarded primarily as a marker of respiratory microbial complexity and a potential indicator of a more prolonged or complicated clinical course, rather than as evidence that each detected microorganism directly contributed to disease severity.

Despite the longer hospitalization and higher complication rate observed among viral–viral and viral–bacterial co-detection patients, the need for low-flow oxygen, HFNC, CPAP, invasive mechanical ventilation, and overall respiratory support did not differ significantly between groups. These findings suggest that disease severity in bronchiolitis cannot be assessed solely by ventilatory requirements. Similar observations have been reported in previous studies, highlighting that coinfections may prolong recovery without necessarily increasing the risk of acute respiratory failure [22]. PICU admission was uncommon in both groups and no significant difference was observed. Given the limited number of events, the study was not powered to detect small differences in intensive care requirements. Moreover, admission to intensive care may be influenced not only by disease severity but also by local organizational factors and clinicians’ decisions regarding monitoring of infants presenting with more complex microbiological profiles. Larger prospective studies using standardized severity scores will be necessary to clarify whether coinfections independently increase the risk of critical illness.

An important finding with potential antimicrobial-stewardship implications was the substantially higher use of antibiotics among infants with viral–bacterial co-detection compared with those with single-virus infection (81.4% vs. 44.9%), as well as compared with viral–viral co-detection (90.9% vs. 53.7%, *p* < 0.001). However, this association should not be interpreted as evidence that bacterial molecular detection represented clinically relevant bacterial infection or directly determined antibiotic prescribing. Bacterial targets detected by PCR in upper-airway specimens, particularly *H. influenzae* and *S. pneumoniae*, may represent nasopharyngeal colonization rather than active bacterial disease. In our retrospective dataset, the determinants of antibiotic prescription could not be systematically reconstructed. Moreover, antibiotic use was not separately analyzed according to viral–viral versus viral–bacterial co-detection, and antibiotic duration and treatment modifications following molecular testing were not systematically recorded. Therefore, although bacterial co-detection was associated with substantially greater antibiotic exposure, our data cannot establish whether multiplex PCR identification of bacterial targets directly influenced antibiotic selection, treatment duration, or subsequent de-escalation. Descriptive treatment data reported in Supplementary File S2 showed differences in antimicrobial selection between the two groups. Amoxicillin–clavulanate was the most frequently prescribed antibiotic in both the single-virus and co-detection groups (27.0% and 50.0%, respectively), followed by clarithromycin (8.0% and 17.0%). Broader-spectrum agents, including meropenem, were used only in a small number of patients in the co-detection group. These differences may reflect greater clinical concern for bacterial infection among infants with co-detection, but cannot be attributed directly to the multiplex PCR results. Nevertheless, infants with viral–bacterial co-detection tended to show higher inflammatory markers, and chest radiography was more frequently performed in this group, with some examinations showing infiltrates or consolidations. These clinical findings may have contributed to the greater use of antibiotics by increasing clinical suspicion of concomitant bacterial infection. However, because these variables were not systematically collected, their relative contribution to antibiotic prescribing could not be formally assessed. More broadly, rapid multiplex molecular testing may have a potential role in antimicrobial stewardship by rapidly identifying a viral etiology and documenting viral co-circulation, thereby supporting a more targeted clinical assessment and potentially reducing unnecessary empirical antibiotic treatment when there are no clinical or laboratory findings suggestive of bacterial infection. Conversely, detection of bacterial targets should not be interpreted in isolation as evidence of bacterial disease, particularly when testing is performed on upper-airway specimens, and should be integrated with clinical findings and other available biomarkers. Thus, the potential stewardship benefit of multiplex PCR is likely to depend not only on rapid pathogen identification but also on appropriate interpretation of molecular results within the clinical context. These findings nevertheless highlight an important antimicrobial-stewardship issue: molecular detection of potentially colonizing bacteria may increase diagnostic uncertainty and antibiotic exposure in children with a predominantly viral respiratory syndrome. Prospective studies integrating standardized clinical criteria, inflammatory biomarkers, bacterial cultures, and radiological findings when clinically indicated are warranted to better distinguish clinically relevant bacterial infection from colonization and to assess the impact of molecular testing on antibiotic prescribing, treatment duration, and de-escalation.

From an epidemiological perspective, our findings confirm the predominant role of RSV throughout the entire study period, both as the leading cause of single-virus bronchiolitis and as the microorganism most frequently identified in co-detections. Rhinovirus represented the second most common viral pathogen, whereas Haemophilus influenzae and Streptococcus pneumoniae were the bacterial species detected most frequently in Viral–Bacterial co-detection patients. These findings are broadly consistent with previous epidemiological studies describing RSV as the principal driver of bronchiolitis hospitalization, while highlighting the frequent coexistence of bacterial respiratory pathogens in children with severe disease [22,23]. The six-year observation period also captured the profound epidemiological changes associated with the COVID-19 pandemic. The apparent increase in the complexity of microbiological profiles observed in the post-pandemic period likely reflects both the renewed simultaneous circulation of multiple respiratory pathogens and the routine implementation of highly sensitive multiplex molecular diagnostic techniques [24].

A particularly relevant aspect of the present study is the evaluation of the first epidemic season following the introduction of universal nirsevimab prophylaxis (2024–2025). The real-world analysis conducted in two birth centers in Western Sicily provides preliminary information regarding the feasibility, safety, and early clinical observations associated with the implementation of universal nirsevimab administration in routine neonatal care. Overall, prophylaxis was administered to 415 of 458 newborns, corresponding to a high uptake of 90.6%, suggesting that integration of nirsevimab into routine neonatal pathways is feasible when supported by structured counselling, appropriate organization, and coordination among healthcare professionals. This uptake was broadly consistent with early European implementation experiences. In Ireland, a national programme reported an uptake of 83.0% during the 2024–2025 RSV season, while a regional implementation programme in the Mid-West achieved a higher uptake of 89.4%; in the latter setting, nirsevimab was administered predominantly before hospital discharge, with 80.9% of newborns receiving prophylaxis within four days of birth [25]. Similarly, a prospective real-world study conducted in a French maternity setting reported an acceptance rate of 91.6%, with antenatal information and counselling contributing to parental acceptance [26]. These figures are best considered implementation benchmarks rather than uniform European targets, as national programmes differ in organization, eligibility criteria, and definitions of uptake or acceptance. Within this context, the 90.6% uptake observed in our cohort compares favourably with these early European experiences, while the variability between our two centers provides an additional operational insight. Uptake reached 94.8% at the Policlinic “Paolo Giaccone” University Hospital in Palermo compared with 83.0% at the “Salvatore Cimino” Hospital in Termini Imerese, with refusal rates of 4.2% and 17.0%, respectively. Although these differences may partly reflect variations in healthcare setting and population characteristics, they also suggest a potential role for local organizational factors and parental counselling pathways in determining implementation success. The higher refusal rate observed among Italian families compared with foreign families (10.0% vs. 4.0%) should be interpreted cautiously, particularly given the limited sample size and the absence of information on potentially relevant sociodemographic and communication-related factors. Nevertheless, these findings emphasize that successful implementation of universal RSV prophylaxis requires not only availability of the medication, but also consistent and effective communication strategies—as previously demonstrated in other pediatric clinical settings [27]—involving neonatologists, pediatricians, general practitioners, obstetricians, and other healthcare professionals engaged in prenatal and postnatal counselling [28]. An important operational lesson emerging from both our findings and other European implementation experiences concerns the timing of administration. Administration during the birth hospitalization and, whenever feasible, before maternity discharge can minimize missed opportunities and ensure timely protection during the early months of life. Embedding nirsevimab administration within established neonatal discharge pathways, supported by antenatal counselling and coordinated communication among maternity, neonatal, and pediatric healthcare professionals, may facilitate consistently high population-level coverage while reducing the need for subsequent outpatient contacts. This approach may be particularly relevant for health systems planning universal RSV prophylaxis, where ensuring timely administration and minimizing differences in uptake across healthcare settings are key implementation challenges. In contrast, previous prevention strategies based on palivizumab were restricted to selected high-risk infants and therefore had a substantially lower impact on the overall population burden of RSV disease [29].

The safety analysis seems to confirm the favorable tolerability profile of nirsevimab observed in randomized clinical trials and early real-world experiences, although the observational design and sample size limit the ability to detect rare adverse events. In our cohort, only 3.1% of treated infants experienced mild and transient adverse events, mainly consisting of local injection-site reactions and low-grade fever, with no cases requiring additional medical evaluation, treatment, or hospitalization. These findings are consistent with pivotal clinical trials and post-marketing surveillance data, in which nirsevimab demonstrated a safety profile comparable to placebo or standard care [30]. In particular, the MELODY trial reported a low frequency of adverse events considered related to nirsevimab, without clinically relevant differences compared with the control group [10], while the MEDLEY study did not identify additional safety concerns in high-risk infants, including preterm newborns and children with underlying medical conditions [31].

When interpreting the clinical outcomes observed in our nirsevimab analysis, it is important to consider that the two participating centers represented different neonatal populations. The University Hospital cohort included a significantly higher proportion of preterm infants and newborns requiring Neonatology or NICU care, reflecting its role as a referral center for high-risk pregnancies and complex neonatal conditions. Conversely, the Termini Imerese cohort mainly included healthy term newborns. Therefore, differences in respiratory outcomes between centers should be interpreted in the context of these different baseline risk profiles rather than as a direct comparison of prophylaxis effectiveness between institutions. Despite the higher proportion of infants theoretically at increased risk in the University Hospital cohort, the overall incidence of respiratory infections was not significantly higher compared with the peripheral birth center. Furthermore, RSV positivity among hospitalized infants was lower in the University Hospital population despite the greater proportion of preterm infants and newborns with potential vulnerability factors. Although these observations cannot demonstrate a causal protective effect, they are compatible with a potential contribution of systematic prophylaxis to reducing the occurrence of RSV-associated disease among prophylaxed infants. Of particular interest, none of the RSV-positive infants identified in our cohort had been born preterm, despite the presence of a relevant proportion of preterm infants among enrolled newborns. Several factors may have contributed to this finding, including greater prioritization of prophylaxis completion among preterm infants, closer clinical surveillance, and increased awareness among healthcare providers regarding RSV prevention in high-risk populations.

The comparison between prophylaxed and non-prophylaxed infants represents one of the clinically relevant aspects of our analysis, providing preliminary real-world observations regarding respiratory outcomes. However, these findings should be interpreted cautiously given the observational study design, the limited number of non-prophylaxed infants, and the differential, clinically indicated respiratory testing. Non-prophylaxed infants showed a higher frequency of respiratory infections and RSV detection than prophylaxed infants. Importantly, the substantially higher testing rate among non-prophylaxed infants limits the interpretation of RSV detection as an estimate of comparative risk or incidence. The lower frequency of RSV detection among nirsevimab-prophylaxed infants is compatible with a potential protective effect of immunoprophylaxis, although this observation should be interpreted cautiously in view of the differential testing pattern, the non-randomized allocation, the limited number of non-prophylaxed infants, and the possibility of residual confounding. Nevertheless, these findings, together with the favorable safety profile and the lower frequency of respiratory complications observed among prophylaxed infants, provide preliminary real-world observations that are directionally consistent with the protective effect of nirsevimab against RSV-associated lower respiratory tract disease demonstrated in randomized and pragmatic clinical trials [10,11], but do not independently establish comparative effectiveness in our cohort. These observations are broadly consistent with the direction of effect reported in clinical trials and recent real-world studies evaluating the effectiveness of nirsevimab in preventing severe RSV disease. The MELODY trial demonstrated that a single dose of nirsevimab significantly reduced medically attended RSV lower respiratory tract infections among healthy late-preterm and term infants, with protection extending throughout the first RSV season [10]. Similarly, the HARMONIE trial, conducted in a pragmatic real-world setting across European countries, showed a significant reduction in RSV-associated hospitalizations and very severe RSV lower respiratory tract infections among infants receiving nirsevimab compared with standard care [11]. More recent real-world implementation studies have confirmed these observations, reporting substantial reductions in RSV-related hospital admissions after the introduction of universal prophylaxis programs [32,33,34,35,36]. Our observations should be interpreted within the limitations of an observational design, as residual confounding related to baseline clinical characteristics, healthcare access, parental acceptance, and differences in clinical management cannot be completely excluded.

The present study also provides relevant information regarding the relationship between respiratory pathogen profiles and clinical outcomes in hospitalized infants with bronchiolitis. Viral–viral and viral–bacterial co-detections represented a frequent finding in our cohort and were associated with longer hospitalization and a higher frequency of complications compared with single-virus infections. Importantly, these associations were not limited to unadjusted comparisons. In the multivariate analyses, respiratory pathogen co-detection remained independently associated with both prolonged hospitalization and complications after adjustment for age at admission, prematurity, underlying comorbidities, and epidemic season. Co-detection was associated with an additional 2.37 days of hospitalization (adjusted β = 2.37, 95% CI: 1.56–3.18; *p* < 0.001) and with more than twice the odds of complications (aOR = 2.15, 95% CI: 1.25–3.69; *p* = 0.006). These findings provide evidence that the association between co-detection and the two main clinical outcomes was not fully explained by the baseline factors included in the models. Nevertheless, the observed associations should not be interpreted as evidence of causality. The retrospective observational design limits causal inference, and residual confounding by factors not included in the models cannot be excluded. Although our stratified univariate analysis (Table 5b) partially mitigated confounding by showing that co-detection remained significantly associated with longer stay and higher complication rates in term infants and in infants without underlying comorbidities, the absence of statistically significant associations in preterm infants and those with comorbidities should be considered in light of the smaller sample sizes and the higher baseline clinical vulnerability of these groups. The multivariate findings therefore strengthen, but do not establish, the hypothesis that respiratory pathogen co-detection is associated with a more prolonged and complicated clinical course. This limitation is particularly relevant because children with more complex clinical presentations may be more likely to undergo extensive microbiological testing, receive broader diagnostic evaluations, or require longer observation periods, potentially influencing the detection of multiple pathogens and the interpretation of their clinical relevance. Furthermore, the identification of bacterial nucleic acids in respiratory samples obtained through molecular techniques cannot always distinguish active infection from upper airway colonization or prolonged shedding after previous exposure. Therefore, multiple pathogen detection should be interpreted primarily as a marker of respiratory microbial complexity and as a potential indicator of a more complex clinical course, rather than as definitive evidence that the detected pathogens directly contributed to disease severity.

Several additional limitations should be considered. First, the retrospective and observational design limits the possibility of establishing causal relationships between microbiological findings and clinical outcomes. Although multivariate regression models were performed and adjusted for age at admission, prematurity, underlying comorbidities, and epidemic season, residual confounding cannot be completely excluded because other potentially relevant clinical or microbiological factors were not included in the models. Second, the monocentric analysis of the present study was conducted in a single hospital setting in Palermo, which may reduce the generalizability of the findings to other healthcare settings and populations. Additional limitations concern microbiological assessment. Although multiplex molecular assays provide high sensitivity for respiratory pathogen detection, they may identify microorganisms that do not necessarily represent active infection. This issue is particularly relevant for bacterial agents commonly colonizing the upper respiratory tract. No specific quantitative PCR threshold was available or applied to distinguish bacterial colonization from active infection. Therefore, bacterial molecular findings were interpreted in conjunction with the clinical and laboratory context and, when indicated and/or available, radiographic findings and conventional bacterial culture. However, quantitative bacterial or viral load measurements were not systematically available, and culture confirmation was not performed in all patients, as conventional microbiological testing was performed according to clinical indication and routine laboratory practice. Information regarding bacterial load, quantitative microbiological parameters, antimicrobial susceptibility, and viral genomic characterization was not available and could have contributed to a better understanding of the biological significance of different pathogen combinations. Furthermore, systematic viral load assessment and long-term follow-up after hospital discharge were not performed, preventing evaluation of the relationship between microbiological characteristics and subsequent respiratory outcomes. Finally, another limitation concerns the exclusion of patients with exclusive bacterial detection. This criterion was based on the predominantly viral nature of bronchiolitis, and was adopted to ensure consistency between the clinical diagnosis and the microbiological definition of the study population. However, this approach precluded direct comparison between patients with isolated bacterial detection and those with viral–bacterial co-detection and may have influenced the estimated burden and clinical interpretation of co-detections. Therefore, our findings should be interpreted as describing microbiological complexity within predominantly viral bronchiolitis rather than the full spectrum of bacterial involvement in infants with lower respiratory tract disease.

The nirsevimab analysis also requires cautious interpretation. The comparison between prophylaxed and non-prophylaxed infants was limited by the relatively small number of non-prophylaxed infants and by the non-randomized nature of prophylaxis administration. Because prophylaxis was offered universally but administered on a voluntary basis, the non-prophylaxed group consisted exclusively of infants whose parents declined immunoprophylaxis, potentially introducing selection bias. Therefore, the observed differences in RSV detection and clinical outcomes should be considered supportive real-world observations rather than direct measures of prophylactic effectiveness. Moreover, nasopharyngeal swabbing was performed according to clinical indication rather than systematically in all infants, resulting in substantially different testing frequencies between groups and potentially introducing ascertainment bias. Therefore, the lower RSV detection rates among prophylaxed infants reported in this study represent the proportion of RSV-positive results among infants who underwent clinically indicated respiratory testing, and should not be interpreted as estimates of RSV incidence or population-level comparative risk. The absence of systematic respiratory testing across the entire cohort precludes calculation of a true incidence rate of RSV infection. In addition, the nirsevimab follow-up analysis was also limited by incomplete follow-up, with information available for 399 of 458 enrolled infants (87.1%). The 59 infants who could not be reached were excluded from follow-up analyses, and the possibility of selection bias cannot therefore be excluded. The comparison of clinical outcomes among hospitalized infants according to nirsevimab prophylaxis status also warrants particular caution. Prophylaxed and non-prophylaxed infants differed significantly in age and, importantly, in the prevalence of comorbidities, with a higher comorbidity burden among non-prophylaxed infants. These baseline differences may have contributed to the observed differences in complication rates, length of hospitalization, and respiratory outcomes, and represent important sources of confounding when comparing prophylaxed and non-prophylaxed infants. In addition, differences in timing of presentation, epidemic season, and other clinical characteristics that could not be fully controlled for may have further influenced these comparisons. Because prophylaxis was not randomly assigned, the observed differences cannot be attributed solely to nirsevimab exposure. Therefore, the lower frequency of complications and shorter hospital stay observed among prophylaxed infants cannot be interpreted as evidence of a causal effect of nirsevimab. Given the observational design, the baseline imbalance between groups, the relatively small sample size, and the limited number of events, these findings should be regarded as descriptive and hypothesis-generating.

Despite these limitations, the observed association between viral–viral and viral–bacterial co-detections and prolonged hospitalization, as well as the increased frequency of complications, suggests that multiple pathogen detection may identify a subgroup of infants with a more complex clinical course. Our subgroup and stratified analyses further support an association between viral–bacterial co-detection and prolonged hospitalization, including among term infants without underlying conditions. Future prospective multicenter studies including standardized severity scores, quantitative microbiological parameters, viral load assessment, and complete patient-level datasets will be necessary to determine whether coinfection represents an independent predictor of prolonged hospitalization, complications, or intensive care requirements.

The present study has, however, several strengths. The retrospective analysis of bronchiolitis epidemiology included a relatively large cohort of hospitalized infants observed over six consecutive epidemic seasons characterized by markedly different epidemiological contexts, including the period before, during, and after the COVID-19 pandemic. The use of multiplex molecular diagnostic techniques allowed detailed characterization of viral and bacterial respiratory profiles and provided insight into the evolution of microbiological patterns over time. In addition, the evaluation of the first season following implementation of universal nirsevimab prophylaxis provides preliminary real-world data in different healthcare settings. Specifically, several findings support the clinical relevance of our observations: the high adherence rate achieved after implementation of universal nirsevimab prophylaxis, the favorable safety profile observed in treated infants, the observed pattern of RSV detection among prophylaxed newborns despite the non-systematic nature of respiratory testing, and the absence of RSV-positive cases among preterm infants in our cohort. Together with available evidence from randomized trials and real-world studies [10,11,30,31,32,33,34,35,36,37,38,39,40,41,42,43], these results support the feasibility of integrating universal RSV immunoprophylaxis into routine neonatal care pathways and highlight the potential role of nirsevimab as an important component of future strategies aimed at reducing the burden of RSV-associated disease.

## 5. Conclusions

This study highlights the clinical relevance of bronchiolitis as a multifactorial condition in hospitalized infants, in which respiratory pathogen co-detections represent a frequent finding and are associated with increased clinical complexity. In particular, viral–viral and viral–bacterial profiles were commonly observed and were associated with longer hospitalization and a higher frequency of complications. However, the presence of multiple detected agents does not necessarily imply a direct causal contribution of each microorganism to disease severity, particularly when bacterial identification is based on molecular respiratory samples. Therefore, co-detection profiles should be interpreted as markers of respiratory microbial complexity that may provide useful prognostic information rather than as independent determinants of clinical outcome. Whether co-detections represent a causal determinant, a marker of disease severity, or a consequence of prolonged viral infection remains uncertain. The high rate of antibiotic prescription observed among viral–bacterial co-detection patients further emphasizes the need for improved diagnostic tools capable of distinguishing true bacterial infection from upper-airway colonization and supporting more appropriate antimicrobial stewardship.

Within the evolving epidemiological landscape of RSV infection, the introduction of nirsevimab represents a major advancement in prevention strategies. Our real-world findings indicate that universal RSV immunoprophylaxis can be effectively implemented across different healthcare settings, achieving high adherence and an excellent safety profile. Although the observational design and limited comparison between prophylaxed and non-prophylaxed infants do not allow definitive estimates of effectiveness, the lower frequency of RSV detection and the favorable clinical profile observed among prophylaxed infants suggest a potential contribution of nirsevimab to reducing RSV-related disease burden in routine clinical practice [10,11,30,31,32,33,34,35,36,37,38,39,40,41,42,43].

Future research should focus on prospective multicenter studies integrating standardized clinical severity scores, detailed microbiological characterization, immunological parameters, and patient-level analyses to better define risk factors associated with complicated bronchiolitis and to clarify the independent contribution of co-infections to clinical outcomes. In parallel, continuous surveillance of real-world RSV immunoprophylaxis programs will be essential to evaluate long-term effectiveness, sustainability, and impact on healthcare systems. The introduction of nirsevimab represents an important shift from the management of severe RSV disease toward proactive prevention, with potential implications for reducing healthcare burden and improving outcomes in early childhood.

## Figures and Tables

**Table 1:** Characteristics of the population according to epidemic season.

Epidemic Season	Total Hospitalized Patients	Single-Virus Bronchiolitis	Co-Detection Bronchiolitis
2019–2020	101	51	50
2020–2021	16	15	1
2021–2022	80	51	29
2022–2023	133	69	64
2023–2024	115	72	43
2024–2025	135	58	77
Total	580	316	264

**Table 2:** Seasonal distribution of viral pathogens in the single-virus bronchiolitis group.

Viral Pathogen	2019–2020	2020–2021	2021–2022	2022–2023	2023–2024	2024–2025	Total *n* (%)
RSV	45	1	27	42	53	40	208 (65.8)
Rhinovirus	0	4	10	9	12	10	45 (14.2)
SARS-CoV-2	0	9	8	4	0	0	21 (6.6)
Human metapneumovirus	2	0	4	9	1	4	20 (6.3)
Influenza A	0	1	0	4	2	3	10 (3.2)
Parainfluenza virus	4	0	2	0	1	0	7 (2.2)
Influenza B	0	0	0	1	1	0	2 (0.6)
Adenovirus	0	0	0	0	2	0	2 (0.6)
Coronavirus NL63	0	0	0	0	0	1	1 (0.3)
Total	51	15	51	69	72	58	316

RSV = respiratory syncytial virus; SARS-CoV-2 = severe acute respiratory syndrome coronavirus 2.

**Table 3:** Viral and bacterial pathogens detected in infants with bronchiolitis and co-detections (*n* = 264).

Viruses	*n* (%)	Bacteria	*n* (%)
RSV	184 (69.7)	*Haemophilus influenzae*	111 (42.0)
Rhinovirus	100 (37.9)	*Streptococcus pneumoniae*	82 (31.1)
Human metapneumovirus	29 (11.0)	*Moraxella catarrhalis*	31 (11.7)
Influenza A	19 (7.2)	*Staphylococcus aureus*	24 (9.1)
Adenovirus	18 (6.8)	*Mycoplasma pneumoniae*	8 (3.0)
Parainfluenza virus	11 (4.2)	*Pseudomonas aeruginosa*	6 (2.3)
SARS-CoV-2	6 (2.3)	*Bordetella pertussis*	4 (1.5)
Influenza B	6 (2.3)	Other bacteria †	5 (1.9)
Other viruses †	8 (3.0)		
Total viral detections *	381	Total bacterial detections *	273

RSV = respiratory syncytial virus; SARS-CoV-2 = severe acute respiratory syndrome coronavirus 2. * Because more than one pathogen could be detected in the same patient, the total number of viral (*n* = 381) and bacterial (*n* = 273) detections exceeds the number of enrolled infants. Percentages refer to the proportion of patients in whom each microorganism was detected. † Other viruses included Coronavirus NL63, Coronavirus OC43, Coronavirus HKU1, and Enterovirus. Other bacteria included *Klebsiella pneumoniae*, *Klebsiella oxytoca*, *Enterobacter kobei*, *Chlamydia pneumoniae*, *Escherichia coli*, and *Enterococcus faecalis*.

**Table 4:** Comparison between patients with single-virus and viral–viral and/or viral–bacterial co-detection bronchiolitis. Continuous variables are expressed as mean ± Standard Deviation (SD) and median (IQR). Categorical variables are reported as counts (%) and analyzed using chi-square test or Fisher’s exact test. Statistical significance was defined as a two-tailed *p*-value < 0.05 (indicated in bold).

Variable	Single-Virus Bronchiolitis (*n* = 316)	Viral–Viral and Viral–Bacterial Co-Detection (*n* = 264)	Odds Ratio (95% CI)	*p*-Value
Male sex, *n* (%)	172 (54.4%)	154 (58.3%)	1.17 (0.84–1.63)	0.390
Age at admission (months)				0.074
—Mean ± SD	4.5 ± 4.2	5.0 ± 4.4	—	0.074
—Median [IQR]	3.0 [1.0–6.0]	4.0 [2.0–8.0]	—	0.074
Length of hospital stay (days)				**<0.001**
—Mean ± SD	6.35 ± 5.21	8.86 ± 7.42	—	**<0.001**
—Median [IQR]	5.0 [4.0–7.0]	7.0 [5.0–10.0]	—	**<0.001**
Complications/associated conditions, *n* (%)	27 (8.5%)	49 (18.6%)	2.44 (1.47–4.04)	**<0.001**
Underlying comorbidities, *n* (%)	40 (12.7%)	34 (12.9%)	1.02 (0.62–1.68)	0.960
Antibiotic therapy, *n* (%)	142 (44.9%)	215 (81.4%)	5.37 (3.65–7.91)	**<0.001**
Non-invasive oxygen support (low-flow oxygen, HFNC, CPAP), *n* (%)	149 (47.2%)	120 (45.5%)	0.93 (0.67–1.30)	0.740
Invasive mechanical ventilation, *n* (%)	5 (1.6%)	4 (1.5%)	0.96 (0.25–3.60)	1.000
PICU admission, *n* (%)	10 (3.2%)	6 (2.3%)	0.71 (0.26–1.97)	0.780

CI: confidence interval; CPAP: continuous positive airway pressure; HFNC: high-flow nasal cannula; IQR: interquartile range; OR: odds ratio; PICU: pediatric intensive care unit; SD: standard deviation.

**Table 6:** Clinical characteristics and outcomes of hospitalized infants according to nirsevimab prophylaxis (2024–2025 season). Continuous variables are expressed as mean ± Standard Deviation (SD) and median (IQR) and compared using the Mann–Whitney U test. Categorical variables are presented as counts (%) and analyzed using the chi-square test (or Yates’ continuity correction)/Fisher’s exact test, reporting Odds Ratios (ORs) and 95% Confidence Intervals (CIs). *p*-values are reported where applicable, in bold if significant. N/A indicates variables for which statistical analysis was not feasible due to the absence of events or the presence of a single event.

Variable	Nirsevimab (*n* = 57)	No Nirsevimab (*n* = 78)	Odds Ratio (95% CI)	*p*-Value
Age at admission (months)				
—Mean ± SD	4.4 ± 3.2	6.2 ± 4.8	—	0.005
—Median [IQR]	3.0 [1.0–5.0]	5.0 [2.0–9.0]	—	
Length of hospital stay (days)				
—Mean ± SD	5.6 ± 2.1	7.0 ± 4.3	—	0.025
—Median [IQR]	5.0 [4.0–6.0]	6.0 [4.0–8.0]	—	
Complications	2 (3.5%)	21 (26.9%)	0.10 (0.02–0.44)	**<0.001**
Underlying comorbidities	7 (12.3%)	28 (35.9%)	0.25 (0.10–0.63)	**<0.001**
Antibiotic therapy	29 (50.9%)	48 (61.5%)	0.65 (0.32–1.30)	0.210
Non-invasive oxygen support	28 (49.1%)	48 (61.5%)	0.60 (0.30–1.21)	0.150
Single-virus bronchiolitis	28 (49.1%)	47 (60.3%)	0.64 (0.32–1.28)	0.190
Co-detection bronchiolitis	29 (50.9%)	31 (39.7%)	1.57 (0.78–3.15)	0.190
Invasive ventilation	0	0	N/A	N/A
PICU admission	0	1	N/A	N/A

CI: confidence interval; IQR: interquartile range; OR: odds ratio; PICU: pediatric intensive care unit; SD: standard deviation.

**Table 7:** Summary of mild and self-limiting adverse effects observed in patients following nirsevimab immunoprophylaxis (*n* = 415).

Adverse Effects	Number of Patients	% of Total (*n* = 415)
Erythema at Injection Site	4	1.0%
Swelling at Injection Site	4	1.0%
Fever	5	1.2%
Total with Adverse Effects	13	3.1%

## Data Availability

The datasets and materials generated and/or analysed during the current study are available from the corresponding author upon reasonable request.

## Supplementary Materials

The following supporting information can be downloaded at https://www.mdpi.com/article/10.3390/children13091189/s1: Supplementary File S1: Detailed Description of Molecular and Conventional Microbiological Diagnostic Methods. File S2: Supplementary Data on Complications, Comorbidities, Pathogen Profiles, Treatments, and Nirsevimab Cohort Characteristics. File S3: Questionnaire for the Assessment of Respiratory Infections Following Nirsevimab Immunoprophylaxis.

## Abbreviations

The following abbreviations are used in this manuscript:

AGA	Appropriate for gestational age
CPAP	Continuous positive airway pressure
COVID-19	Coronavirus disease 2019
EUCAST	European Committee on Antimicrobial Susceptibility Testing
GDPR	General Data Protection Regulation
HFNC	High-flow nasal cannula
IQR	Interquartile range
LGA	Large for gestational age
NICU	Neonatal intensive care unit
PCR	Polymerase chain reaction
PICU	Pediatric intensive care unit
RSV	Respiratory syncytial virus
SARS-CoV-2	Severe acute respiratory syndrome coronavirus 2
SD	Standard deviation
SGA	Small for gestational age
