# Supplementary material for: Respiratory Pathogen Co-Detection in Hospitalized Infants with Bronchiolitis and Implementation of Universal Nirsevimab Immunoprophylaxis: A Six-Year Observational Study
Source: Children (Basel). 2026 Sep 3;13(9):1189. doi: 10.3390/children13091189 (PMC13605864; doi:10.3390/children13091189)
Supplement: Supplementary file 1 [file children-13-01189-s001.zip › children-4510235-supplementary.pdf]

Supplementary File S1: Detailed Description of Molecular and Conventional Microbiological Diagnostic Methods

The Allplex™ Respiratory Full Panel Assay (Seegene Inc., South Korea) is a multiplex real-time reverse transcription polymerase chain reaction (RT-PCR) assay designed for the simultaneous detection and identification of 26 respiratory pathogens, including viral and bacterial agents, based on a one-step real-time RT-PCR platform. Clinically significant respiratory infections caused by a wide range of viral and bacterial pathogens are common in both adult and pediatric populations. Early and accurate identification of the causative agents, including in cases of coinfection, is essential to guide appropriate therapeutic strategies and support infection control measures. Conventional diagnostic approaches are often time-consuming and may lack sensitivity and comprehensiveness, frequently leading to empirical treatment without etiological confirmation. The assay detects 16 respiratory viruses (influenza B; respiratory syncytial virus [RSV] A and B; adenovirus; enterovirus; human metapneumovirus; parainfluenza viruses 1–4; bocavirus; coronaviruses 229E, NL63, and OC43; rhinovirus), 7 bacterial pathogens (*Bordetella pertussis/parapertussis, Chlamydophila pneumoniae, Haemophilus influenzae, Legionella pneumophila, Mycoplasma pneumoniae, Streptococcus pneumoniae*), and 3 influenza A subtypes (H1, H1pdm09, and H3). The system is fully automatable from nucleic acid extraction to PCR setup, with a total turnaround time of approximately 4 hours and 30 minutes and a throughput ranging from 20 to 94 samples per run depending on configuration. Samples were not tested in duplicate, and rapid antigen detection tests were not used in the present study. The multiplex PCR assay was used as a qualitative molecular detection method. Cycle threshold (Ct) values or other quantitative measures of bacterial or viral load were not used for clinical classification and were not available for retrospective analysis. Therefore, no assay-specific quantitative threshold was applied to distinguish bacterial colonization from active infection. Bacterial molecular findings were interpreted in conjunction with clinical, laboratory, radiographic, and culture findings when available. In particular, detection of bacterial targets commonly associated with upper-airway colonization, such as Haemophilus influenzae or Streptococcus pneumoniae, was considered potentially compatible with colonization and was not regarded as definitive evidence of bacterial disease in the absence of supporting clinical or laboratory findings. Pathogen-specific analytical performance characteristics, including sensitivity and specificity, vary according to the individual target and assay configuration; therefore, no single overall sensitivity or specificity value was considered applicable to the multiplex panel as a whole.

In addition to molecular testing, conventional microbiological culture was performed on respiratory specimens (nasopharyngeal swabs and/or hypopharyngeal aspirates) when clinically indicated or when sufficient sample volume was available. For bacterial isolation, specimens were inoculated onto standard solid media routinely used in clinical microbiology laboratories, including 5% sheep blood agar, chocolate agar, and MacConkey agar. Plates were incubated at 35–37°C for 18–24 hours under aerobic conditions, while chocolate agar plates were incubated in a 5% CO_2-enriched atmosphere to support the growth of fastidious organisms. When no growth was observed after 24 hours and clinical suspicion persisted, incubation was extended up to 48 hours. Plates were examined daily for colony morphology, hemolysis patterns, and growth characteristics. Bacterial identification was performed using conventional phenotypic and biochemical methods (including catalase, oxidase, coagulase testing, and carbohydrate utilization profiles) and/or automated identification systems available during the study period. These included matrix-assisted laser desorption/ionization time-of-flight mass spectrometry (MALDI-TOF MS; Bruker Daltonics, Bremen, Germany and/or bioMérieux,

Marcy-l'Étoile, France) and automated biochemical identification platforms (e.g., VITEK 2 system, bioMérieux, Marcy-l'Étoile, France), depending on laboratory availability. Organisms were identified at species level whenever possible, whereas group-level identification was used for closely related respiratory pathogens when necessary.

Antimicrobial susceptibility testing (AST) was performed on clinically relevant bacterial isolates using automated systems and/or standardized disk diffusion methods, according to laboratory workflow and organism characteristics. Interpretation of susceptibility results followed the European Committee on Antimicrobial Susceptibility Testing (EUCAST) breakpoints applicable during the study period.

Where feasible, molecular and culture-based methods were applied to the same specimen to enhance diagnostic yield and to allow comparison between nucleic acid detection (including potentially non-viable organisms identified by PCR) and viable bacterial growth. Discordant results were interpreted in the clinical context, particularly in cases of polymicrobial detection or suspected colonization.

Supplementary File S2: Supplementary Data on Complications, Comorbidities, Pathogen Profiles, Treatments, and Nirsevimab Cohort Characteristics

Table S1. Distribution of complications and bronchiolitis-associated conditions across six epidemic seasons (N = 76 patients, 13% of the total cohort)

Epidemic Season	Seasonal Cohort (N)	Patients with Complications, n (%)	Observed Complications / Associated Conditions
2019–2020	100	16 (16.0%)	Pneumonia (n = 6, including 2 with atelectasis), acute respiratory failure (n = 3), gastroenteritis (n = 3), pleuritis (n = 1), UTI (n = 1), pericarditis (n = 1), supraglottic laryngitis (n = 1)
2020–2021	17	1 (6.0%)	UTI (n = 1)
2021–2022	82	9 (11.0%)	Pneumonia (n = 6), acute respiratory failure (n = 1), gastroenteritis (n = 1), gingivostomatitis (n = 1)
2022–2023	133	18 (13.5%)	Pneumonia (n = 9), acute respiratory failure (n = 6), pneumonia with pneumothorax (n = 1), UTI (n = 1), seizure (n = 1)
2023–2024	113	9 (8.0%)	Acute respiratory failure (n = 3), pneumonia (n = 2), gastroenteritis (n = 2), scabies (n = 1)

Epidemic Season	Seasonal Cohort (*N*)	Patients with Complications, n (%)	Observed Complications / Associated Conditions
2024–2025	144	23 (16.0%)	Pneumonia ($n = 8$, including 1 aspiration pneumonia), bronchopneumonia ($n = 6$), gastroenteritis ($n = 2$), cyanotic/apneic episode ($n = 1$), supraglottic laryngitis ($n = 1$), seizure ($n = 1$), sepsis ($n = 1$), heart failure with acute renal injury ($n = 1$), pleuritis with neutropenia and *C. difficile* infection ($n = 1$)
Total	580	76 (13.0%)	—

Abbreviations: UTI, Urinary Tract Infection.

Table S2. Prevalence and spectrum of underlying comorbidities identified in hospitalized infants ($N = 74$, 13.0% of cohort)

Organ System / Category	Specific Comorbidity	Patients, *n*
Cardiovascular	Congenital Heart Diseases (CHD)*	18
Gastrointestinal / Surgical	Gastroesophageal Reflux Disease (GERD)	6
	Inguinal hernia	2
	History of esophageal atresia repair	1
	History of congenital diaphragmatic hernia repair	1
Respiratory / Airway	Laryngomalacia	1
	Ex-preterm with chronic lung disease	1
	Pneumatocele	1
Neurological / Developmental	Hypotonia	6
	Neurodevelopmental delay / Encephalopathy	2
	Epileptic encephalopathy	1

Organ System / Category	Specific Comorbidity	Patients, *n*
Infectious (Congenital)	Congenital Cytomegalovirus (CMV)	4
	Congenital Toxoplasmosis	1
Allergic / Immunological	Cow's Milk Protein Allergy (CMPA)	5
	Inhalant allergies	3
	Congenital neutropenia	1
Genetic / Syndromic	Down syndrome	2
	Cornelia de Lange syndrome	1
	Klinefelter syndrome	1
Renal / Urological	Hydronephrosis	4
	Hypospadias	1
Hematological	Iron-deficiency anemia	2
Other	Congenital hip dysplasia	1
	Unilateral hearing loss	1
Total Patients with Comorbidities	—	74 (13.0%)

**Breakdown of Congenital Heart Diseases ($n = 18$): Patent Foramen Ovale (PFO, $n = 6$); Pulmonary Stenosis ($n = 3$); Ventricular Septal Defect (VSD, $n = 2$); Atrial Septal Defect (ASD, $n = 2$); Intermediate AV Canal ($n = 1$); ASD + VSD ($n = 1$); PFO + Patent Ductus Arteriosus ($n = 1$); Atrial Septal Aneurysm ($n = 1$); Aortic Insufficiency with Bicuspid Aorta ($n = 1$).*

Table S3. Vaccination status across the entire study cohort ($N = 580$)

Vaccination Status	Number of Patients (n)	Percentage (%)
Up to date	200	34.5%
Not vaccinated	201	34.7%
Delayed	22	3.8%
Unknown	157	27.1%

Vaccination Status	Number of Patients (n)	Percentage (%)
Total	580	100.0%

Table S4. Seasonal distribution of viral pathogens detected in single-virus infections ($N = 320$)

Viral Pathogen	2019–2020 (*n* = 51)	2020–2021 (*n* = 15)	2021–2022 (*n* = 51)	2022–2023 (*n* = 69)	2023–2024 (*n* = 72)	2024–2025 (*n* = 58)
RSV	45 (88.2%)	1 (6.7%)	27 (52.9%)	42 (60.9%)	53 (73.6%)	40 (69.0%)
Rhinovirus	0 (0.0%)	4 (26.7%)	10 (19.6%)	9 (13.0%)	12 (16.7%)	10 (17.2%)
hMPV	2 (3.9%)	0 (0.0%)	4 (7.8%)	9 (13.0%)	1 (1.4%)	4 (6.9%)
Influenza A	0 (0.0%)	1 (6.7%)	0 (0.0%)	4 (5.8%)	2 (2.8%)	3 (5.2%)
Influenza B	0 (0.0%)	0 (0.0%)	0 (0.0%)	1 (1.4%)	1 (1.4%)	0 (0.0%)
Parainfluenza	4 (7.8%)	0 (0.0%)	2 (3.9%)	0 (0.0%)	1 (1.4%)	0 (0.0%)
SARS-CoV-2	0 (0.0%)	9 (60.0%)	8 (15.7%)	4 (5.8%)	0 (0.0%)	0 (0.0%)
Adenovirus	0 (0.0%)	0 (0.0%)	0 (0.0%)	0 (0.0%)	2 (2.8%)	0 (0.0%)
Coronavirus NL63	0 (0.0%)	0 (0.0%)	0 (0.0%)	0 (0.0%)	0 (0.0%)	1 (1.7%)
Total	51 (100.0%)	15 (100.0%)	51 (100.0%)	69 (100.0%)	72 (100.0%)	58 (100.0%)

Abbreviations: hMPV, Human Metapneumovirus; RSV, Respiratory Syncytial Virus.

Table S5. Comparison of medical therapies and supportive interventions in single-virus vs. co-detection bronchiolitis cases

Treatment/Supportive Intervention Category	Single-virus bronchiolitis (*N* = 320), *n* (%)	Co-detection bronchiolitis (*N* = 264), *n* (%)
**Antimicrobials & Antifungals**		

**Treatment/Supportive Intervention Category**	**Single-virus bronchiolitis (*N* = 320), *n* (%)**	**Co-detection bronchiolitis (*N* = 264), *n* (%)**
Amoxicillin–Clavulanic Acid	86 (26.9%)	133 (50.4%)
Clarithromycin	26 (8.1%)	46 (17.4%)
Ceftriaxone	19 (5.9%)	27 (10.2%)
Cefotaxime	20 (6.3%)	12 (4.5%)
Sulfamethoxazole–Trimethoprim	1 (0.3%)	7 (2.7%)
Cefixime	3 (0.9%)	5 (1.9%)
Meropenem	0 (0.0%)	5 (1.9%)
Cefaclor	1 (0.3%)	4 (1.5%)
Azithromycin	1 (0.3%)	1 (0.4%)
Chloramphenicol	0 (0.0%)	1 (0.4%)
Gentamicin	0 (0.0%)	1 (0.4%)
Acyclovir	0 (0.0%)	1 (0.4%)
Nystatin	10 (3.1%)	16 (6.1%)
**Corticosteroid Therapy**		
Aerosolized Beclomethasone	213 (66.6%)	202 (76.5%)
Systemic Betamethasone	184 (57.5%)	193 (73.1%)
Systemic Methylprednisolone	69 (21.6%)	85 (32.2%)
Bronchodilators & Inhalation Therapy		
Aerosolized Salbutamol	137 (42.8%)	163 (61.7%)
3% Hypertonic Saline aerosol	40 (12.5%)	29 (11.0%)
Aerosolized Adrenaline	12 (3.8%)	9 (3.4%)
**Supportive & Respiratory Support**		
Low-flow Oxygen Therapy	134 (41.9%)	111 (42.0%)

Treatment/Supportive Intervention Category	Single-virus bronchiolitis (*N* = 320), *n* (%)	Co-detection bronchiolitis (*N* = 264), *n* (%)
Intravenous Rehydration	110 (34.4%)	93 (35.2%)
High-Flow Nasal Cannula (HFNC)	14 (4.4%)	8 (3.0%)
Invasive Mechanical Ventilation	5 (1.6%)	4 (1.5%)
Nasal CPAP (n-CPAP)	1 (0.3%)	1 (0.4%)

Table S6. Spectrum of complications, associated conditions, and comorbidities stratified by infection group

Category	Clinical Condition	Single-virus (*N* = 40), *n*	Co-detection (*N* = 49), *n*
Complications/Associated Conditions	Pneumonia/Bronchopneumonia	8	27
	Acute Respiratory Failure	8	5
	Enteritis/Gastroenteritis	1	8
	Urinary Tract Infection (UTI)	2	1
	Seizures	2	0
	Pulmonary Atelectasis	1	1
	Pleuritis	1	1
	Supraglottic Laryngitis	1	1
	Cyanosis/Apnea episodes	0	2
	Pericarditis	1	0
	Heart Failure with Acute Renal Injury	1	0
	Scabies	1	0
	Pneumothorax (PNX)	0	1
	Sepsis	0	1

Category	Clinical Condition	Single-virus (N = 40), n	Co-detection (N = 49), n
	Gingivostomatitis	0	1
Underlying Comorbidities	Congenital Heart Disease (CHD)	17	9
	Hydronephrosis	0	4
	Gastroesophageal Reflux Disease (GERD)	3	2
	Cow's Milk Protein Allergy (CMPA)	3	2
	Neurodevelopmental Delay/Encephalopathy	1	2
	Down Syndrome	0	2
	Hypotonia	0	2
	Growth Delay	0	2
	Inhalant Allergy	3	0

Table S7. Seasonal breakdown and pattern analysis of co-detection cases ($N = 264$)

Season (n Co-detections)	Subtype Category	Pathogen Combination Profile (n)
2019–2020 (n = 50)	Viral–Viral (n = 5)	RSV + Influenza A (n = 3), RSV + Rhinovirus (n = 1), hMPV + Influenza A (n = 1)
	Viral–Bacterial (n = 45)	RSV + *H. influenzae* (n = 12), RSV + *S. pneumoniae* (n = 7), RSV + *S. aureus* + *H. influenzae* (n = 4), RSV + *H. influenzae* + *S. pneumoniae* (n = 4), RSV + *S. aureus* (n = 3), RSV + *M. catarrhalis* (n = 3), Influenza A + *H. influenzae* (n = 2), RSV + Influenza A + *S. aureus* (n = 2), RSV + Influenza A + *H. influenzae* + *S. pneumoniae* (n = 2), RSV + Influenza A + *H. influenzae* (n = 1), RSV + Enterovirus + *H. influenzae* + *S. pneumoniae* (n = 1), RSV + *H. influenzae* + *M. catarrhalis* (n = 1),

Season (n Co-detections)	Subtype Category	Pathogen Combination Profile (n)
		Influenza A + *H. influenzae* + *S. pneumoniae* ($n = 1$), RSV + *P. aeruginosa* ($n = 1$), RSV + *H. influenzae* + *Candida albicans* ($n = 1$)
2020–2021 ($n = 1$)	Viral–Bacterial ($n = 1$)	RSV + Rhinovirus + *S. pneumoniae* + *S. aureus* ($n = 1$)
2021–2022 ($n = 29$)	Viral–Viral ($n = 6$)	RSV + Rhinovirus ($n = 5$), SARS-CoV-2 + Rhinovirus ($n = 1$)
	Viral–Bacterial ($n = 23$)	RSV + *S. pneumoniae* ($n = 4$), RSV + *H. influenzae* ($n = 3$), RSV + *S. aureus* ($n = 2$), RSV + Rhinovirus + *S. pneumoniae* ($n = 2$), RSV + *M. catarrhalis* ($n = 2$), hMPV + *H. influenzae* ($n = 2$), Rhinovirus + *M. catarrhalis* ($n = 1$), RSV + *H. influenzae* + *S. pneumoniae* ($n = 1$), RSV + hMPV + *M. catarrhalis* ($n = 1$), Rhinovirus + Adenovirus + *S. pneumoniae* + *S. aureus* + *H. influenzae* + *P. aeruginosa* ($n = 1$), Rhinovirus + hMPV + *S. pneumoniae* + *M. catarrhalis* ($n = 1$), Parainfluenza + *M. catarrhalis* ($n = 1$), hMPV + *S. pneumoniae* ($n = 1$)
2022–2023 ($n = 64$)	Viral–Viral ($n = 27$)	RSV + Rhinovirus ($n = 14$), hMPV + Rhinovirus ($n = 5$), Rhinovirus + Parainfluenza ($n = 1$), Rhinovirus + SARS-CoV-2 ($n = 1$), RSV + Influenza A ($n = 1$), RSV + Rhinovirus + Adenovirus ($n = 1$), RSV + Adenovirus ($n = 1$), RSV + hMPV ($n = 1$), RSV + SARS-CoV-2 ($n = 1$), hMPV + Rhinovirus + Adenovirus ($n = 1$)
	Viral–Bacterial ($n = 37$)	RSV + *H. influenzae* ($n = 4$), RSV + Rhinovirus + *H. influenzae* ($n = 3$), hMPV + *H. influenzae* ($n = 2$), hMPV + Rhinovirus + *H. influenzae* + *M. catarrhalis* ($n = 2$), RSV + Influenza A + *E. faecalis* ($n = 2$), Rhinovirus + *H. influenzae* ($n = 2$), RSV + *S. pneumoniae* + *M. catarrhalis* ($n = 1$), RSV + *M. catarrhalis* + *P. aeruginosa* ($n = 1$), Rhinovirus + Influenza A + *M. catarrhalis* + *S. pneumoniae* + *H. influenzae* ($n = 1$), RSV + Rhinovirus + *S. pneumoniae* ($n = 1$), RSV + *S. pneumoniae* + *P. aeruginosa* ($n = 1$), RSV + *S. pneumoniae* + *S. aureus* ($n = 1$), hMPV + *M. catarrhalis* + *S. pneumoniae* ($n = 1$), RSV + *H. influenzae* + *M. catarrhalis* ($n = 1$), RSV + Rhinovirus + SARS-CoV-2 + *H. influenzae* ($n = 1$), Rhinovirus + *S. pneumoniae* + *S. aureus* + *H. influenzae* ($n = 1$), RSV + hMPV + *H. influenzae* ($n = 1$), hMPV + *S. aureus* ($n = 1$), RSV + Rhinovirus + *S. pneumoniae* + *H. influenzae* ($n = 1$), RSV + *S. aureus* ($n = 1$), Rhinovirus + *S. pneumoniae* ($n = 1$), Influenza B + *H. influenzae* ($n = 1$), Rhinovirus + Parainfluenza + *H. influenzae* ($n = 1$), hMPV + *M. catarrhalis* ($n = 1$), Rhinovirus + *M. catarrhalis* ($n = 1$), Rhinovirus + *S. pneumoniae* + *M. catarrhalis* ($n = 1$), Rhinovirus + *H. influenzae* + *S. aureus* ($n = 1$), RSV + *K. pneumoniae* ($n = 1$)
2023–2024 ($n = 43$)	Viral–Viral ($n = 11$)	RSV + Rhinovirus ($n = 3$), RSV + SARS-CoV-2 ($n = 2$), RSV + Coronavirus OC43 ($n = 2$), Rhinovirus + Influenza A ($n = 1$), RSV +

Season (*n* Co-detections)	Subtype Category	Pathogen Combination Profile (*n*)
		Rhinovirus + Adenovirus ($n = 1$), RSV + Adenovirus ($n = 1$), Parainfluenza + Rhinovirus ($n = 1$)
	Viral–Bacterial ($n = 32$)	RSV + *H. influenzae* ($n = 6$), RSV + *S. pneumoniae* + *H. influenzae* ($n = 4$), RSV + *M. catarrhalis* ($n = 3$), Rhinovirus + *S. pneumoniae* + *H. influenzae* ($n = 2$), Rhinovirus + *H. influenzae* ($n = 2$), Rhinovirus + *M. catarrhalis* ($n = 1$), Rhinovirus + *S. pneumoniae* + *M. catarrhalis* ($n = 1$), Rhinovirus + *S. pneumoniae* ($n = 1$), Rhinovirus + Coronavirus OC43 + *B. pertussis* ($n = 1$), Rhinovirus + Parainfluenza + *S. pneumoniae* ($n = 1$), Parainfluenza + *M. catarrhalis* + *P. aeruginosa* ($n = 1$), Parainfluenza + *S. aureus* ($n = 1$), Parainfluenza + *S. pneumoniae* + *H. influenzae* ($n = 1$), RSV + *B. pertussis* ($n = 1$), RSV + *S. pneumoniae* ($n = 1$), RSV + *S. aureus* ($n = 1$), RSV + Rhinovirus + *B. pertussis* ($n = 1$), RSV + Rhinovirus + *M. catarrhalis* + *S. aureus* + *H. influenzae* ($n = 1$), RSV + Adenovirus + *B. pertussis* ($n = 1$), Adenovirus + *M. catarrhalis* ($n = 1$)
2024–2025 ($n = 77$)	Viral–Viral ($n = 18$)	RSV + Rhinovirus ($n = 12$), RSV + Adenovirus ($n = 2$), RSV + Rhinovirus + Coronavirus NL63 ($n = 1$), Adenovirus + Rhinovirus ($n = 1$), Influenza B + Coronavirus NL63 ($n = 1$), hMPV + Rhinovirus ($n = 1$)
	Viral–Bacterial ($n = 59$)	RSV + *S. pneumoniae* ($n = 7$), RSV + *S. pneumoniae* + *H. influenzae* ($n = 6$), RSV + Adenovirus + *S. pneumoniae* + *H. influenzae* ($n = 4$), Rhinovirus + *H. influenzae* ($n = 3$), Rhinovirus + *S. pneumoniae* ($n = 2$), Rhinovirus + *S. pneumoniae* + *H. influenzae* ($n = 3$), Rhinovirus + Adenovirus + *S. pneumoniae* + *H. influenzae* ($n = 2$), RSV + *M. pneumoniae* + *S. pneumoniae* + *H. influenzae* ($n = 2$), RSV + *H. influenzae* ($n = 2$), RSV + Rhinovirus + *S. pneumoniae* + *H. influenzae* ($n = 2$), RSV + Rhinovirus + *H. influenzae* ($n = 2$), RSV + Influenza B + *H. influenzae* ($n = 2$), hMPV + *S. pneumoniae* ($n = 2$), hMPV + *M. pneumoniae* ($n = 2$), Rhinovirus + *S. aureus* ($n = 1$), Rhinovirus + *E. coli* ($n = 1$), Rhinovirus + *S. aureus* (MRSA) + *E. kobei* + *K. oxytoca* ($n = 1$), Rhinovirus + Parainfluenza + *M. pneumoniae* ($n = 1$), Rhinovirus + Coronavirus NL63 + *M. pneumoniae* ($n = 1$), RSV + *M. pneumoniae* ($n = 1$), RSV + *M. catarrhalis* ($n = 1$), RSV + *S. pneumoniae* + *H. influenzae* + *S. aureus* ($n = 1$), RSV + Influenza A + *H. influenzae* ($n = 1$), RSV + Influenza B + *M. pneumoniae* ($n = 1$), RSV + Coronavirus HKV1 + *S. pneumoniae* ($n = 1$), Parainfluenza + *S. pneumoniae* + *H. influenzae* ($n = 1$), Influenza A + Influenza B + *H. influenzae* ($n = 1$), Influenza A + *H. influenzae* ($n = 1$), hMPV + *H. influenzae* ($n = 1$), hMPV + *M. catarrhalis* + *H. influenzae* ($n = 1$), hMPV + *S. pneumoniae* + *H. influenzae* ($n = 1$), hMPV + *C. pneumoniae* ($n = 1$)

Figure S1. Pathogen interplay and co-detection profiles among infants hospitalized with bronchiolitis (N = 264). **(A)** Frequency distribution of predominant viral–viral and viral–bacterial pathogen combinations across six epidemic seasons (2019–2025). Dual-viral detection was dominated by RSV + Rhinovirus (n = 47), whereas viral–bacterial cases were primarily driven by RSV in combination with *Haemophilus influenzae* (n = 31) and/or *Streptococcus pneumoniae* (n = 19). **(B)** Heatmap matrix representing pairwise pathogen co-occurrences, illustrating strong interactions between primary respiratory viruses (RSV, Rhinovirus, Metapneumovirus) and secondary bacterial colonizers.

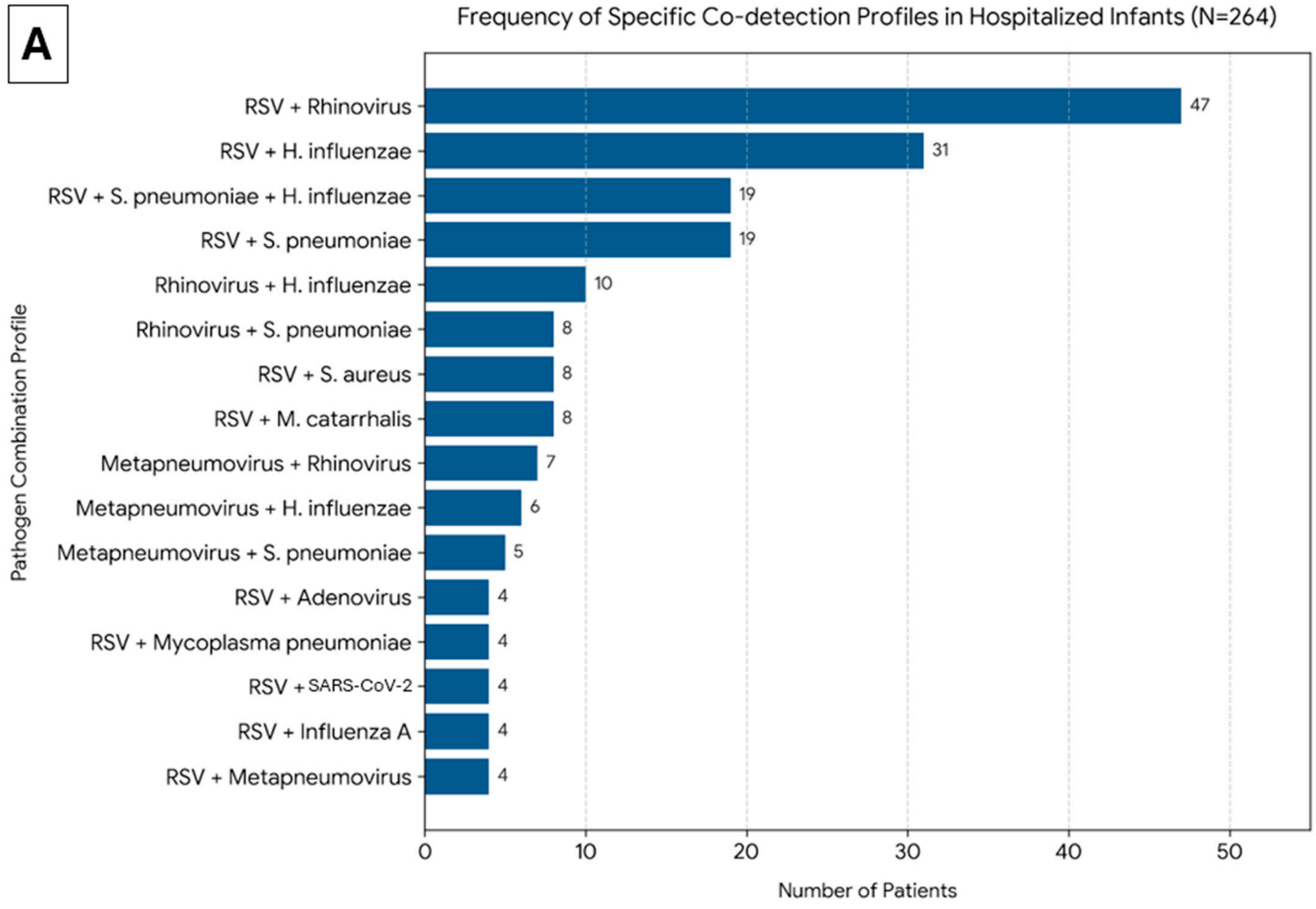

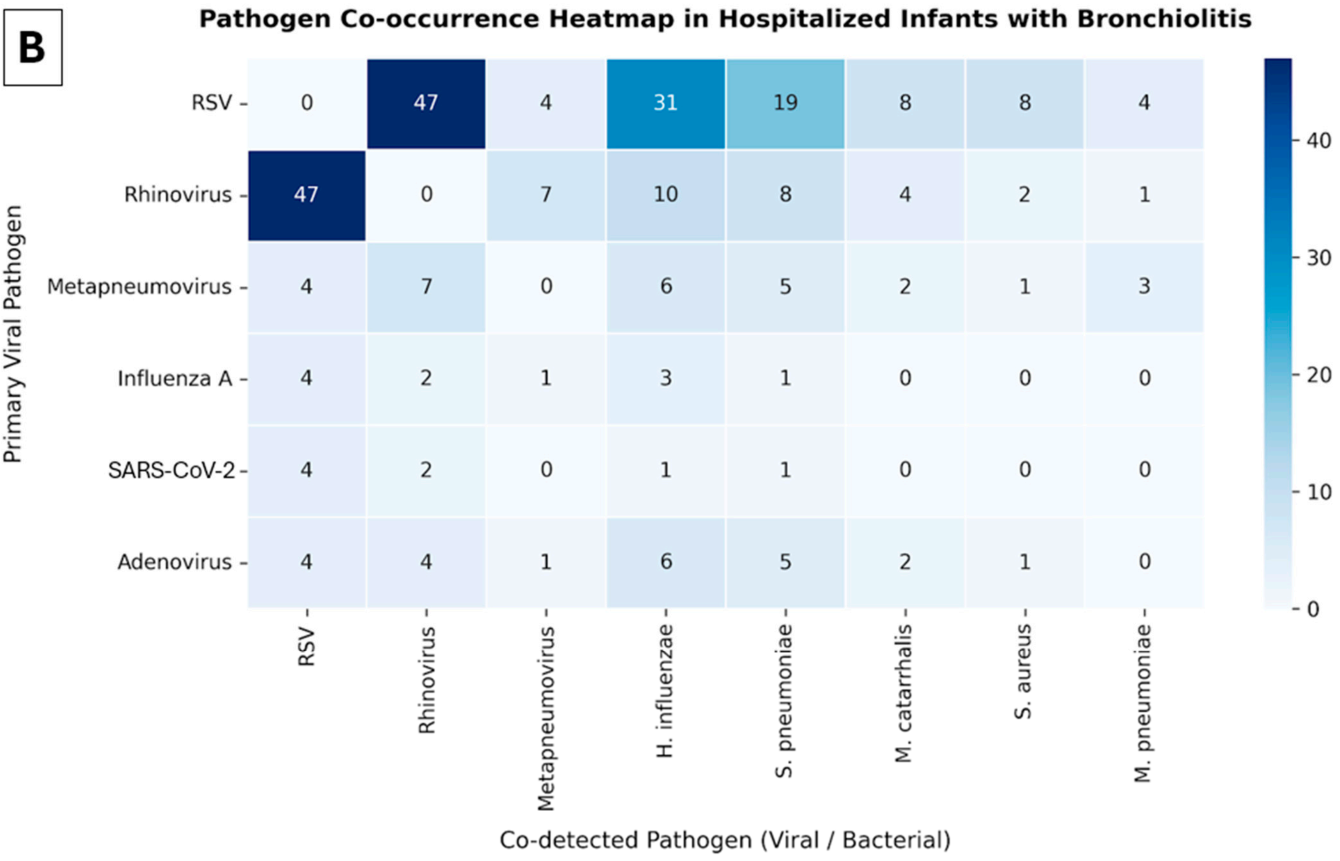

Table S8. Sex distribution of infants hospitalized for bronchiolitis according to nirsevimab immunoprophylaxis status (2024–2025 season, $N = 135$)

Sex	Nirsevimab received (n = 57), n (%)	Nirsevimab not received (N = 78), n (%)	Total (N = 135), n (%)
**Male**	37 (64.9%)	41 (52.6%)	78 (57.8%)
**Female**	20 (35.1%)	37 (47.4%)	57 (42.2%)

Table S9. Gestational age classifications by clinical center in the nirsevimab cohort (N = 456)

Study Center	AGA, n (%)	LGA, n (%)	SGA, n (%)	Total Patients (N)
"Paolo Giaccone" University Hospital, Palermo	236 (81.4%)	20 (6.9%)	34 (11.7%)	290
"Salvatore Cimino" Hospital, Termini Imerese	136 (81.9%)	12 (7.2%)	18 (10.8%)	166
**Total Cohort**	**372 (81.6%)**	**32 (7.0%)**	**52 (11.4%)**	**456**

Supplementary File S3: Questionnaire for the Assessment of Respiratory Infections Following Nirsevimab Immunoprophylaxis

Questionnaire assessing bronchiolitis risk after nirsevimab prophylaxis, including prophylaxis status, side effects, respiratory infections, therapy and hospitalization, pathogen detection, oxygen therapy, feeding type, and sibling information.

#	Question	Response Options
1	Has your child received immunoprophylaxis with the monoclonal antibody nirsevimab (Beyfortus)?	○ Yes ○ No
2	Were any side effects reported after nirsevimab administration?	○ No side effects ○ Fever ○ Redness at injection site ○ Swelling at injection site
3	Has your child experienced any respiratory infections?	○ No ○ Common cold ○ Cough and/or fever ○ Bronchiolitis ○ Pneumonia
4	In which period did your child become ill? (please indicate the month)	______________________
5	How was your child's respiratory infection managed?	○ No pediatric visit needed ○ Visited pediatrician – home therapy ○ Emergency Department – discharged with home therapy ○ Emergency Department – hospitalized ○ Admitted to Intensive Care Unit

6	What therapy was administered?	○ Nasal washes only ○ Nasal washes and aerosol therapy ○ Aerosol therapy and corticosteroids ○ Aerosol therapy, corticosteroids, and antibiotics
7	Indicate the hospital where the admission took place (if applicable)	______________________
8	Was a nasopharyngeal swab performed by the pediatrician or in the hospital?	○ Yes ○ No
9	If a swab was performed, which pathogen was detected?	○ RSV (respiratory syncytial virus) ○ Rhinovirus ○ Influenza virus ○ Metapneumovirus ○ SARS-CoV-2
10	Did your child require oxygen therapy?	○ No ○ Yes, required oxygen in hospital
11	What type of feeding does your child receive?	○ Breast milk ○ Formula milk ○ Mixed feeding
12	Does your child have older siblings?	○ Yes ○ No
13	How many older brothers and/or sisters does your child have?	______________________
